# Supplementary material for: The Impact of Social Determinants of Health on Meningococcal Vaccination Awareness, Delivery, and Coverage in Adolescents and Young Adults in the United States: A Systematic Review
Source: Vaccines (Basel). 2023 Jan 24;11(2):256. doi: 10.3390/vaccines11020256 (PMC9962690; doi:10.3390/vaccines11020256)
Supplement: Supplementary file 1 [file vaccines-11-00256-s001.zip › vaccines-2135308-supplementary.pdf]

Supplementary Material

Table S-1. Electronic Database Search Strategies

Table S-1a. MEDLINE Search Strategy (Conducted 10 June 2022)

Limits: humans; English; no comments, letters, editorials; US only

Search no.	Search terms
**Disease**	
1	exp Meningococcal Infections/ or exp Neisseria meningitidis/ or meningiti*.tw. or meningococc*.tw. or Micrococcus intracellularis.tw. or Neisseria weichselbaumi.tw. or IMD.tw.
**Vaccination**	
2	exp Vaccination/ or Vaccines/ or exp Immunization/ or exp Immunization Programs/ or vaccin*.tw. or immuni*.tw.
3	1 and 2
4	exp Meningococcal Vaccines/ or (acwy vax or meningococcal vacci* or antimeningococcal vacci* or bexsero or mc 4 vacci* or mc4 vacci* or MCP CRM197 conjugate vacci* or men quad tt or menactra or menacwy or menafrivac or menbvac or mencevax or meningitec or meningoccal vacci* or meningococcal group B vacci* or meningococcus vacci* or meninvact or menjugate or menomune or menpovax or menquadfi or menveo or menzb or neisvac c or New Zealand candidate vacci* or nimenrix or nonamen or tetramen-t or trumenba or va mengoc bc).tw.
5	3 or 4
**Health equity**	
6	exp Health Equity/ or exp Healthcare Disparities/ OR exp Health Status Disparities/ OR (health* adj3 (disparit* or inequalit* or inequit* or equit* or equalit*)).tw.
7	5 and 6
8	exp Minority Health/ or exp "health disparity, minority and vulnerable populations"/ or "ethnic and racial minorities"/ or hierarchy, social/ or social marginalization/ or social vulnerability/ or exp "Social Determinants of Health"/ or Minority Health.tw. or vulnerable*.tw. or disadvantaged.tw. or under served.tw. or underserved.tw. or neglect*.tw. or marginal*.tw. or socially excluded.tw. or social deprivation.tw. or social disparit*.tw. or social determinant*.tw. or social inequit*.tw. or racial disparit*.tw. or racial inequit*.tw. or racial equalit*.tw. or racial equity.tw. or ethnic disparit*.tw. or ((ethnic* or minorit* or race* or racial) adj3 (background or communit* or group* or population*)).tw. or (high-risk adj3 (communit* or group* or population* or people or person*)).tw. or exp African Americans/ or african american*.tw. or african ancestry.tw. or exp African Continental Ancestry Group/ or aian.tw. or exp American Native Continental Ancestry Group/ or Asian Continental Ancestry Group/ or asian*.tw. or black*1.tw. or caucasian*1.tw. or ghetto*1.tw. or hispanic*1.tw. or indian*1.tw. or latino*1.tw. or latina*1.tw. or native american*1.tw. or oceanic ancestry group/ or pacific islander*1.tw. or people of color.tw. or people of colour.tw. or slum*1.tw. or white.tw. or whites.tw.
9	5 and 8
10	exp "Emigrants and Immigrants"/ or emigrant*.tw. or Immigrant*.tw. or migrant*.tw. or exp Refugees/ or refugee*.tw. or Asylum Seeker*.tw. or displaced person*.tw.
11	5 and 10
12	exp Poverty/ or exp poverty area/ or exp Medically Underserved Area/ or hierarchy, social/ or exp socioeconomic factors/ or poverty.tw. or low income*.tw. or middle

Search no.	Search terms
	income*.tw. or low resource*.tw. or impoverish.tw. or indigence.tw. or indigency.tw. or exp Homeless Persons/ or homeless.tw. or jobless.tw. or job seek*.tw. or single parent*.tw. or single mother*.tw. or lone parent*.tw. or health insurance status.tw. or (socio economic* adj3 (inequalit* or unequal* or inequit* or disparit* or imparit* or discrepanc* or imbalance* or factor*)).tw.
13	5 and 12
14	exp "Sexual and Gender Minorities"/ or ((Sexual or Gender) adj2 Minorit*).tw. or homosexual*.tw. or homo sexual*.tw. or bisexual*.tw. or gay.tw. or gays.tw. or lesbian*.tw. or pansexual*.tw. or queer*.tw. or asexual*.tw. or transgender*.tw. or transsexual*.tw. or intersex*.tw. or gender-expansive.tw. or LGBT*.tw. or GLBTI*.tw. or 2 spirit*.tw. or gender diverse.tw. or gender fluid*.tw. or non binary.tw. or men who have sex with men.tw.
15	5 and 14
16	exp sociodemographic factors/ or exp community resources/ or exp Health Services Accessibility/ or "Health Services Needs and Demand"/ or exp health services, indigenous/ or exp rural health/ or exp rural health services/ or exp urban health/ or exp urban health services/ or exp rural population/ or exp urban population/ or ((geographic or regional or urban or rural or demographic or cultural) adj4 (inequalit* or unequal* or inequit* or disparit* or imparit* or discrepanc* or imbalance* or barrier* or facilitat* or motivat*)).tw. or rural area*.tw. or rural population*.tw. or rural health*.tw. or urban health*.tw. or urban population.tw.
17	5 and 16
18	((improv* or enhanc* or encourag* or increas* or support* or assist* or maximi* or promot* or facilitat*) adj3 (uptake or cover* or acceptanc* or complianc* or complet* or acceptab* or adoption or rate* or access or equit* or equalit*)).tw.
19	5 and 18
20	((decreas* or reduc* or minimi* or detect* or chang*) adj3 (inequalit* or unequal* or inequit* or disparit* or imparit* or discrepanc* or imbalance*)).tw.
21	5 and 20
22	exp Anti-Vaccination Movement/ or Anti-Vaccination.tw. or anti-VAC.tw. or anti-vaccine.tw. or anti-vax*.tw. or antivaccinal.tw. or antivaccination.tw. or antivaccine.tw. or antivax*.tw.
23	5 and 22
24	exp Vaccination Hesitancy/ or ((vaccin* or immuni*) adj3 (hesitan* or refus* or delay* or barrier* or obstacle* or restrict* or obstruct* or impede* or restrain* or constrain* or inhibit*)).tw.
25	5 and 24
26	exp Health Promotion/ or ((vaccin* or immuni*) adj3 (equit* or confidence or complet* or compliance or comply or complie* or adher* or access* or facilitat* or uptake or up take or take up or improve* or enhance* or encourag* or support* or promot* or optimiz* or optimis* or motivat* or accept* or incentive* or attend* or adopt* or persuad* or persuasion or attitud* or know* or awareness* or affordab*)).tw.
27	5 and 26
28	exp Physician Incentive Plans/ or exp Reimbursement, Incentive/ or exp Medical Audit/ or exp Clinical Audit/ or incentive plan*.tw. or reimbursement*.tw. or medical audit*.tw. or clinical audit*.tw. or ((pediatric* or parent* or gp or general practic* or physician* or doctor* or clinician* or nurse practitioner* or midwife or midwives) adj3 (attitud* or know* or awareness*)).tw. or ((vaccin* or immuni* or parent* or household* or house-hold* or clinical) adj3 decision-making).tw.
29	5 and 28
30	Exp Health Behavior/ or exp Unsafe Sex/ or ((unprotect* or condomless or risk or high-risk) adj3 (sex* or intercourse*)).tw. or (sex* adj2 (behavior* or behaviour*)).tw.
31	5 and 30

Search no.	Search terms
32	exp Alcoholism/ or alcoholism.tw. or (alcohol* adj2 (dependenc* or addiction* or abuse or intoxication* or use*)).tw.
33	5 and 32
34	exp Drug Misuse/ or exp illicit drugs/ or ((drug* or substance* or non prescription* or nonprescription*) adj2 (misuse or abuse or addiction* or dependenc* or use*)).tw.
35	5 and 34
36	exp smoking/ or smoking.tw. or smoker*.tw.
37	5 and 36
38	Exp Religion/ or religion*.tw. or religious.tw. or prayer*.tw. or spirituality.tw. or theology.tw. or Mysticism.tw. or buddhism.tw. or christian*.tw. or confucianism.tw. or hinduism.tw. or islam*.tw. or judaism.tw. or mormonism.tw. or shamanism.tw. or taoism.tw.
39	5 and 38
40	exp educational status/ or (educational adj3 status).tw. or literacy.tw. or illiteracy.tw. or (academic adj2 fail*).tw.
41	5 and 40
42	exp Social Justice/ or exp Social Values/ or exp Resource Allocation/ or exp Health Care Rationing/ or exp Health Priorities/es or equity.tw.
43	5 and 42
44	7 or 9 or 11 or 13 or 15 or 17 or 19 or 21 or 23 or 25 or 27 or 29 or 31 or 33 or 35 or 37 or 39 or 41 or 43
**Exclusions**	
45	(animals/ not humans/) or exp Animals, Laboratory/ or exp Animal Experimentation/ or exp Models, Animal/ or exp Rodentia/ or (rat or rats or mouse or mice).ti.
46	Comment/ or letter/ or editorial/ or Case Reports/ or news/ or exp historical article/ or Anecdotes as Topic/ or (letter or comment*).ti.
**Total**	
47	44 not (45 or 46)
48	limit 47 to english language
49	exp united states/ or united states.ti,ab. or usa.ti,ab. or united states.in. or usa.in. or us.in. or alabama.in. or alaska.in. or arizona.in. or arkansas.in. or california.in. or colorado.in. or connecticut.in. or delaware.in. or florida.in. or georgia.in. or hawaii.in. or idaho.in. or illinois.in. or indiana.in. or iowa.in. or kansas.in. or kentucky.in. or louisiana.in. or maine.in. or maryland.in. or massachusetts.in. or michigan.in. or minnesota.in. or mississippi.in. or missouri.in. or montana.in. or nebraska.in. or nevada.in. or new hampshire.in. or new jersey.in. or new mexico.in. or new york.in. or north carolina.in. or north dakota.in. or ohio.in. or oklahoma.in. or oregon.in. or pennsylvania.in. or rhode island.in. or south carolina.in. or south dakota.in. or tennessee.in. or texas.in. or utah.in. or vermont.in. or virginia.in. or washington.in. or west virginia.in. or wisconsin.in. or wyoming.in. or puerto rico.in. or alabama.mp. or alaska.mp. or arizona.mp. or arkansas.mp. or california.mp. or colorado.mp. or connecticut.mp. or delaware.mp. or florida.mp. or georgia.mp. or hawaii.mp. or idaho.mp. or illinois.mp. or indiana.mp. or iowa.mp. or kansas.mp. or kentucky.mp. or louisiana.mp. or maine.mp. or maryland.mp. or massachusetts.mp. or michigan.mp. or minnesota.mp. or mississippi.mp. or missouri.mp. or montana.mp. or nebraska.mp. or nevada.mp. or new hampshire.mp. or new jersey.mp. or new mexico.mp. or new york.mp. or north carolina.mp. or north dakota.mp. or ohio.mp. or oklahoma.mp. or oregon.mp. or pennsylvania.mp. or rhode island.mp. or south carolina.mp. or south dakota.mp. or tennessee.mp. or texas.mp. or utah.mp. or vermont.mp. or virginia.mp. or washington.mp. or west virginia.mp. or wisconsin.mp. or wyoming.mp. or puerto rico.mp.
50	48 and 49

Table S-1b. Embase Search Strategy (Conducted 10 June 2022)

Limits: Humans; English; No Comments, letters, editorials or case reports. US only. Abstracts 2020-2022

Search No.	Search Terms
**Disease**	
#1	'meningococcosis'/exp OR 'neisseria meningitidis'/exp OR meningiti*:ti,ab OR meningococc*:ti,ab OR 'micrococcus intracellularis':ti,ab OR 'neisseria weichselbaumi':ti,ab OR imd:ti,ab
**Vaccination**	
#2	'vaccination'/exp OR 'vaccine'/de OR 'immunization'/exp OR 'preventive health service'/exp OR vaccin*:ti,ab OR immuni*:ti,ab
#3	#1 AND #2
#4	'meningococcus vaccine'/exp OR 'acwy vax':ti,ab OR 'meningococcal vacci*':ti,ab OR 'antimeningococcal vacci*':ti,ab OR bexsero:ti,ab OR 'mc 4 vacci*':ti,ab OR 'mc4 vacci*':ti,ab OR 'mcp crm197 conjugate vacci*':ti,ab OR 'men quad tt':ti,ab OR menactra:ti,ab OR menacwy:ti,ab OR menafrivac:ti,ab OR menbvac:ti,ab OR mencevax:ti,ab OR meningitec:ti,ab OR 'meningoccal vacci*':ti,ab OR 'meningococcal group b vacci*':ti,ab OR 'meningococcus vacci*':ti,ab OR meninvact:ti,ab OR menjugate:ti,ab OR menomune:ti,ab OR menpovax:ti,ab OR menquadfi:ti,ab OR menveo:ti,ab OR menzb:ti,ab OR 'neisvac c':ti,ab OR 'new zealand candidate vacci*':ti,ab OR nimenrix:ti,ab OR nonamen:ti,ab OR 'tetramen-t':ti,ab OR trumenba:ti,ab OR 'va mengoc bc':ti,ab
#5	#3 OR #4
#6	'health equity'/exp OR 'health care disparity'/exp OR 'health disparity'/exp OR ((health* NEAR/3 (disparit* OR inequalit* OR inequit* OR equit* OR equalit*)):ti,ab)
#7	#5 AND #6
#8	'minority health'/exp OR 'vulnerable population'/exp OR 'ethnic group'/exp OR 'social hierarchy'/exp OR 'social exclusion'/exp OR 'social vulnerability'/de OR 'social determinants of health'/exp OR 'minority health':ti,ab OR vulnerable*:ti,ab OR disadvantaged:ti,ab OR 'under served':ti,ab OR underserved:ti,ab OR neglect*:ti,ab OR marginal*:ti,ab OR 'socially excluded':ti,ab OR 'social deprivation':ti,ab OR 'social disparit*':ti,ab OR 'social determinant*':ti,ab OR 'social inequit*':ti,ab OR 'racial disparit*':ti,ab OR 'racial inequit*':ti,ab OR 'racial equalit*':ti,ab OR 'racial equity':ti,ab OR 'ethnic disparit*':ti,ab OR (((ethnic* OR minorit* OR race* OR racial) NEAR/3 (background OR communit* OR group* OR population*)):ti,ab) OR (('high risk' NEAR/3 (communit* OR group* OR population* OR people OR person*)):ti,ab) OR 'black person'/exp OR 'african american*':ti,ab OR 'african ancestry':ti,ab OR aian:ti,ab OR 'american indian'/exp OR 'asian continental ancestry group'/exp OR asian*:ti,ab OR black*:ti,ab OR caucasian*:ti,ab OR ghetto*:ti,ab OR hispanic*:ti,ab OR indian*:ti,ab OR latino*:ti,ab OR latina*:ti,ab OR 'native american*':ti,ab OR 'oceanic ancestry group'/exp OR 'pacific islander*':ti,ab OR 'people of color':ti,ab OR 'people of colour':ti,ab OR slum*:ti,ab OR white:ti,ab OR whites:ti,ab
#9	#5 AND #8
#10	'migrant'/exp OR emigrant*:ti,ab OR immigrant*:ti,ab OR migrant*:ti,ab OR 'refugee'/exp OR refugee*:ti,ab OR 'asylum seeker*':ti,ab OR 'displaced person*':ti,ab
#11	#5 AND #10

Search No.	Search Terms
#12	'poverty'/exp OR 'health care planning'/exp/mj OR 'socioeconomics'/exp/mj OR poverty:ti,ab OR 'low income*':ti,ab OR 'middle income*':ti,ab OR 'low resource*':ti,ab OR impoverish:ti,ab OR indigence:ti,ab OR indigency:ti,ab OR 'homeless person'/exp OR homeless:ti,ab OR jobless:ti,ab OR 'job seek*':ti,ab OR 'single parent*':ti,ab OR 'single mother*':ti,ab OR 'lone parent*':ti,ab OR 'health insurance status':ti,ab OR (('socio economic*' NEAR/3 (inequalit* OR unequal* OR inequit* OR disparit* OR imparit* OR discrepanc* OR imbalance* OR factor*)):ti,ab)
#13	#5 AND #12
#14	'sexual and gender minority'/exp OR (((sexual OR gender) NEAR/2 minorit*):ti,ab) OR homosexual*:ti,ab OR 'homo sexual*':ti,ab OR bisexual*:ti,ab OR gay:ti,ab OR gays:ti,ab OR lesbian*:ti,ab OR pansexual*:ti,ab OR queer*:ti,ab OR asexual*:ti,ab OR transgender*:ti,ab OR transsexual*:ti,ab OR intersex*:ti,ab OR 'gender-expansive':ti,ab OR lgbt*:ti,ab OR glbti*:ti,ab OR '2 spirit*':ti,ab OR 'gender diverse':ti,ab OR 'gender fluid*':ti,ab OR 'non binary':ti,ab OR 'men who have sex with men':ti,ab
#15	#5 AND #14
#16	'sociodemographics'/exp OR 'community resource'/exp OR 'health care access'/exp OR 'health service'/de OR 'indigenous health care'/exp OR 'rural health'/exp OR 'rural health care'/exp OR 'urban health'/exp OR 'rural population'/exp OR 'urban population'/exp OR (((geographic OR regional OR urban OR rural OR demographic OR cultural) NEAR/4 (inequalit* OR unequal* OR inequit* OR disparit* OR imparit* OR discrepanc* OR imbalance* OR barrier* OR facilitat* OR motivat*)):ti,ab) OR 'rural area*':ti,ab OR 'rural population*':ti,ab OR 'rural health*':ti,ab OR 'urban health*':ti,ab OR 'urban population*':ti,ab
#17	#5 AND #16
#18	((improv* OR enhanc* OR encourag* OR increas* OR support* OR assist* OR maximi* OR promot* OR facilitat*) NEAR/3 (uptake OR cover* OR acceptanc* OR complianc* OR complet* OR acceptab* OR adoption OR rate* OR access OR equit* OR equalit*)):ti,ab
#19	#5 AND #18
#20	((decreas* OR reduc* OR minimi* OR detect* OR chang*) NEAR/3 (inequalit* OR unequal* OR inequit* OR disparit* OR imparit* OR discrepanc* OR imbalance*)):ti,ab
#21	#5 AND #20
#22	'anti-vaccination movement'/exp OR 'anti-vaccination':ti,ab OR 'anti-vac':ti,ab OR 'anti-vaccine*':ti,ab OR 'anti-vax*':ti,ab OR antivaccinal:ti,ab OR antivaccination:ti,ab OR antivaccine*:ti,ab OR antivax*:ti,ab
#23	#5 AND #22
#24	'vaccine hesitancy'/exp OR (((vaccin* OR immuni*) NEAR/3 (hesitan* OR refus* OR delay* OR barrier* OR obstacle* OR restrict* OR obstruct* OR impede* OR restrain* OR constrain* OR inhibit*)):ti,ab)
#25	#5 AND #24
#26	'health promotion'/exp OR (((vaccin* OR immuni*) NEAR/3 (equit* OR confidence OR complet* OR compliance OR comply OR complie* OR adher* OR access* OR facilitat* OR uptake OR 'up take' OR 'take up' OR improve* OR enhance* OR encourag* OR support* OR promot* OR optimiz* OR optimis* OR motivat* OR accept* OR incentive* OR attend* OR adopt* OR persuad* OR persuasion OR attitud* OR know* OR awareness* OR affordab*)):ti,ab)
#27	#5 AND #26
#28	'personnel management'/exp OR 'reimbursement'/exp OR 'clinical audit'/exp OR 'incentive plan*':ti,ab OR reimbursement*:ti,ab OR 'medical audit*':ti,ab OR 'clinical audit*':ti,ab OR (((pediatric* OR parent* OR gp OR 'general practic*' OR physician* OR doctor* OR clinician* OR 'nurse practitioner*' OR midwife OR midwives) NEAR/3 (attitud* OR know* OR awareness*)):ti,ab) OR (((vaccin* OR

Search No.	Search Terms
	immuni* OR parent* OR household* OR 'house-hold*' OR clinical) NEAR/3 'decision making'):ti,ab)
#29	#5 AND #28
#30	'health behavior'/exp OR 'unsafe sex'/exp OR (((unprotect* OR condomless OR risk OR 'high-risk' OR unsafe) NEAR/3 (sex* OR intercourse*)):ti,ab) OR ((sex* NEAR/2 (behavior* OR behaviour*)):ti,ab)
#31	#5 AND #30
#32	'alcoholism'/exp OR alcoholism:ti,ab OR ((alcohol* NEAR/2 (dependenc* OR addiction* OR abuse OR intoxication* OR use*)):ti,ab)
#33	#5 AND #32
#34	'drug misuse'/exp OR 'illicit drug'/exp OR (((drug* OR substance* OR 'non prescription*' OR nonprescription*) NEAR/2 (misuse OR abuse OR addiction* OR dependenc* OR use*)):ti,ab)
#35	#5 AND #34
#36	'smoking'/exp OR smoking:ti,ab OR smoker*:ti,ab
#37	#5 AND #36
#38	'religion'/exp OR religion*:ti,ab OR religious:ti,ab OR prayer*:ti,ab OR spirituality:ti,ab OR theology:ti,ab OR mysticism:ti,ab OR buddhism:ti,ab OR christian*:ti,ab OR confucianism:ti,ab OR hinduism:ti,ab OR islam*:ti,ab OR judaism:ti,ab OR mormonism:ti,ab OR shamanism:ti,ab OR taoism:ti,ab
#39	#5 AND #38
#40	'educational status'/exp OR ((educational NEAR/3 status):ti,ab) OR literacy:ti,ab OR illiteracy:ti,ab OR ((academic NEAR/2 fail*):ti,ab)
#41	#5 AND #40
#42	'social justice'/exp OR 'social value'/exp OR 'resource allocation'/exp OR equity:ti,ab OR 'health priorit*':ti,ab OR 'health care rationing':ti,ab
#43	#5 AND #42
#44	#7 OR #9 OR #11 OR #13 OR #15 OR #17 OR #19 OR #21 OR #23 OR #25 OR #27 OR #29 OR #31 OR #33 OR #35 OR #37 OR #39 OR #41 OR #43
#45	'animal'/exp NOT 'human'/exp OR 'experimental animal'/exp OR 'animal experiment'/exp OR 'animal model'/exp OR 'rodent'/exp OR rat:ti OR rats:ti OR mouse:ti OR mice:ti
#46	[editorial]/lim OR [letter]/lim OR 'case report'/exp OR letter:ti OR comment*:ti
**Total**	
#47	#44 NOT (#45 OR #46)
#48	#44 NOT (#45 OR #46) AND [english]/lim AND [embase]/lim
#49	'united states'/exp OR 'united states':ti,ab OR usa:ti,ab OR 'u.s.a.':ti,ab OR 'u.s.':ti,ab OR 'united states':ad OR 'usa':ad OR 'u.s.a.':ad OR 'u.s.':ad OR 'us':ad OR alabama:ad OR alaska:ad OR arizona:ad OR arkansas:ad OR california:ad OR colorado:ad OR connecticut:ad OR delaware:ad OR florida:ad OR georgia:ad OR hawaii:ad OR idaho:ad OR illinois:ad OR indiana:ad OR iowa:ad OR kansas:ad OR kentucky:ad OR louisiana:ad OR maine:ad OR maryland:ad OR massachusetts:ad OR michigan:ad OR minnesota:ad OR mississippi:ad OR missouri:ad OR montana:ad OR nebraska:ad OR nevada:ad OR 'new hampshire':ad OR 'new jersey':ad OR 'new mexico':ad OR 'new york':ad OR 'north carolina':ad OR 'north dakota':ad OR ohio:ad OR oklahoma:ad OR oregon:ad OR pennsylvania:ad OR 'rhode island':ad OR 'south carolina':ad OR 'south dakota':ad OR tennessee:ad OR texas:ad OR utah:ad OR vermont:ad OR virginia:ad OR washington:ad OR 'west virginia':ad OR wisconsin:ad OR wyoming:ad OR 'puerto rico':ad OR alabama:de,ab,ti OR alaska:de,ab,ti OR arizona:de,ab,ti OR arkansas:de,ab,ti OR california:de,ab,ti OR colorado:de,ab,ti OR connecticut:de,ab,ti OR delaware:de,ab,ti OR florida:de,ab,ti OR georgia:de,ab,ti OR hawaii:de,ab,ti OR idaho:de,ab,ti OR illinois:de,ab,ti OR indiana:de,ab,ti OR iowa:de,ab,ti OR kansas:de,ab,ti OR kentucky:de,ab,ti OR louisiana:de,ab,ti OR maine:de,ab,ti OR maryland:de,ab,ti OR massachusetts:de,ab,ti OR michigan:de,ab,ti OR minnesota:de,ab,ti OR mississippi:de,ab,ti OR missouri:de,ab,ti OR montana:de,ab,ti OR nebraska:de,ab,ti OR nevada:de,ab,ti OR

Search No.	Search Terms
	'new hampshire':de,ab,ti OR 'new jersey':de,ab,ti OR 'new mexico':de,ab,ti OR 'new york':de,ab,ti OR 'north carolina':de,ab,ti OR 'north dakota':de,ab,ti OR ohio:de,ab,ti OR oklahoma:de,ab,ti OR oregon:de,ab,ti OR pennsylvania:de,ab,ti OR 'rhode island':de,ab,ti OR 'south carolina':de,ab,ti OR 'south dakota':de,ab,ti OR tennessee:de,ab,ti OR texas:de,ab,ti OR utah:de,ab,ti OR vermont:de,ab,ti OR virginia:de,ab,ti OR washington:de,ab,ti OR 'west virginia':de,ab,ti OR wisconsin:de,ab,ti OR wyoming:de,ab,ti OR 'puerto rico':de,ab,ti
#50	#48 AND #49
#51	#50 NOT ([conference abstract]/lim OR [conference paper]/lim OR [conference review]/lim)
#52	#50 AND ([conference abstract]/lim OR [conference paper]/lim OR [conference review]/lim) AND [2020-2022]/py

Table S-1c. Cochrane Search Strategy (Conducted 10 June 2022)

Limits: Humans; English; No Comments, letters, editorials, case reports or conference abstracts. US only

Search No.	Search Terms
**Disease**	
#1	[mh "Meningococcal Infections"] OR [mh "Neisseria meningitidis"] OR meningiti*:ti,ab OR meningococc*:ti,ab OR "Micrococcus intracellularis":ti,ab OR "Neisseria weichselbaumi":ti,ab OR IMD:ti,ab
**Vaccination**	
#2	[mh Vaccination] OR [mh ^Vaccines] OR [mh Immunization] OR [mh "Immunization Programs"] OR vaccin*:ti,ab OR immuni*:ti,ab
#3	#1 AND #2
#4	[mh "Meningococcal Vaccines"] OR "acwy vax":ti,ab OR (meningococcal NEXT vacci*):ti,ab OR (antimeningococcal NEXT vacci*):ti,ab OR bexsero:ti,ab OR ("mc 4" NEXT vacci*):ti,ab OR (mc4 NEXT vacci*):ti,ab OR ("MCP CRM197 conjugate" NEXT vacci*):ti,ab OR "men quad tt":ti,ab OR menactra:ti,ab OR menacwy:ti,ab OR menafrivac:ti,ab OR menbvac:ti,ab OR mencevax:ti,ab OR meningitec:ti,ab OR (meningoccal NEXT vacci*):ti,ab OR ("meningococcal group B" NEXT vacci*):ti,ab OR (meningococcus NEXT vacci*):ti,ab OR meninvact:ti,ab OR menjugate:ti,ab OR menomune:ti,ab OR menpovax:ti,ab OR menquadfi:ti,ab OR menveo:ti,ab OR menzb:ti,ab OR "neisvac c":ti,ab OR ("New Zealand candidate" NEXT vacci*):ti,ab OR nimenrix:ti,ab OR nonamen:ti,ab OR "tetramen t":ti,ab OR trumenba:ti,ab OR "va mengoc bc":ti,ab
#5	#3 OR #4
#6	[mh "Health Equity"] OR [mh "Healthcare Disparities"] OR [mh "Health Status Disparities"] OR (health* NEAR/3 (disparit* or inequalit* or inequit* or equit* or equalit*)):ti,ab
#7	#5 AND #6
#8	[mh "Minority Health"] OR [mh "health disparity, minority and vulnerable populations"] OR [mh "ethnic and racial minorities"] OR [mh "hierarchy, social"] OR [mh "social marginalization"] OR [mh "social vulnerability"] OR [mh "Social Determinants of Health"] OR "Minority Health":ti,ab OR vulnerable*:ti,ab OR disadvantaged:ti,ab OR "under served":ti,ab OR underserved:ti,ab OR neglect*:ti,ab OR marginal*:ti,ab OR "socially excluded":ti,ab OR "social deprivation":ti,ab OR (social NEXT disparit*):ti,ab OR (social NEXT determinant*):ti,ab OR (social NEXT inequit*):ti,ab OR (racial NEXT disparit*):ti,ab OR (racial NEXT inequit*):ti,ab OR (racial NEXT equalit*):ti,ab OR (racial NEXT equity:ti,ab) OR (ethnic NEXT disparit*):ti,ab OR ((ethnic* OR minorit* OR race* OR racial) NEAR/3 (background OR communit* OR group* OR population*)):ti,ab OR ("high risk" NEAR/3 (communit* OR group* OR population* OR people OR person*)):ti,ab OR [mh "African Americans"] OR (african NEXT american*):ti,ab OR "african ancestry":ti,ab OR [mh "African Continental Ancestry Group"] OR aian:ti,ab OR [mh "American Native Continental Ancestry Group"] OR [mh "Asian Continental Ancestry Group"] OR asian*:ti,ab OR black*:ti,ab OR caucasian*:ti,ab OR ghetto*:ti,ab OR hispanic*:ti,ab OR indian*:ti,ab OR latino*:ti,ab OR latina*:ti,ab OR (native NEXT american*):ti,ab OR [mh "oceanic ancestry group"] OR (pacific NEXT islander*):ti,ab OR "people of color":ti,ab OR "people of colour":ti,ab OR slum*:ti,ab OR white:ti,ab OR whites:ti,ab
#9	#5 AND #8
#10	[mh "Emigrants and Immigrants"] OR emigrant*:ti,ab OR Immigrant*:ti,ab OR migrant*:ti,ab OR [mh Refugees] OR refugee*:ti,ab OR (Asylum NEXT Seeker*):ti,ab OR (displaced NEXT person*):ti,ab
#11	#5 AND #10
#12	[mh Poverty] OR [mh "poverty area"] OR [mh "Medically Underserved Area"] OR [mh "socioeconomic factors"] OR poverty:ti,ab OR (low NEXT income*):ti,ab OR

Search No.	Search Terms
	(middle NEXT income*):ti,ab OR (low NEXT resource*):ti,ab OR impoverish:ti,ab OR indigence:ti,ab OR indigency:ti,ab OR [mh "Homeless Persons"] OR homeless:ti,ab OR jobless:ti,ab OR (job NEXT seek*):ti,ab OR (single NEXT parent*):ti,ab OR (single NEXT mother*):ti,ab OR (lone NEXT parent*):ti,ab OR "health insurance status":ti,ab OR ((socio NEXT economic*) NEAR/3 (inequalit* OR unequal* OR inequit* OR disparit* OR imparit* OR discrepanc* OR imbalance* OR factor*)):ti,ab
#13	#5 AND #12
#14	[mh "Sexual and Gender Minorities"] OR ((Sexual OR Gender) NEAR/2 Minorit*):ti,ab OR homosexual*:ti,ab OR (homo NEXT sexual*):ti,ab OR bisexual*:ti,ab OR gay:ti,ab OR gays:ti,ab OR lesbian*:ti,ab OR pansexual*:ti,ab OR queer*:ti,ab OR asexual*:ti,ab OR transgender*:ti,ab OR transsexual*:ti,ab OR intersex*:ti,ab OR "gender expansive":ti,ab OR LGBT*:ti,ab OR GLBTI*:ti,ab OR (2 NEXT spirit*):ti,ab OR "gender diverse":ti,ab OR (gender NEXT fluid*):ti,ab OR "non binary":ti,ab OR "men who have sex with men":ti,ab
#15	#5 AND #14
#16	[mh "sociodemographic factors"] OR [mh "community resources"] OR [mh "Health Services Accessibility"] OR [mh "Health Services Needs and Demand"] OR [mh "health services, indigenous"] OR [mh "rural health"] OR [mh "rural health services"] OR [mh "urban health"] OR [mh "urban health services"] OR [mh "rural population"] OR [mh "urban population"] OR ((geographic OR regional OR urban OR rural OR demographic OR cultural) NEAR/4 (inequalit* OR unequal* OR inequit* OR disparit* OR imparit* OR discrepanc* OR imbalance* OR barrier* OR facilitat* OR motivat*)):ti,ab OR (rural NEXT area*):ti,ab OR (rural NEXT population*):ti,ab OR (rural NEXT health*):ti,ab OR (urban NEXT health*):ti,ab OR (urban NEXT population*):ti,ab
#17	#5 AND #16
#18	((improv* OR enhanc* OR encourag* OR increas* OR suppORt* OR assist* OR maximi* OR promot* OR facilitat*) NEAR/3 (uptake OR cover* OR acceptanc* OR complianc* OR complet* OR acceptab* OR adoption OR rate* OR access OR equit* OR equalit*)):ti,ab
#19	#5 AND #18
#20	((decreas* OR reduc* OR minimi* OR detect* OR chang*) NEAR/3 (inequalit* OR unequal* OR inequit* OR disparit* OR imparit* OR discrepanc* OR imbalance*)):ti,ab
#21	#5 AND #20
#22	[mh "Anti-Vaccination Movement"] OR "Anti Vaccination":ti,ab OR "anti VAC":ti,ab OR "anti vaccine":ti,ab OR (anti NEXT vax*):ti,ab OR antivaccinal:ti,ab OR antivaccination:ti,ab OR antivaccine:ti,ab OR antivax*:ti,ab
#23	#5 AND #22
#24	[mh "Vaccination Hesitancy"] OR ((vaccin* OR immuni*) NEAR/3 (hesitan* OR refus* OR delay* OR barrier* OR obstacle* OR restrict* OR obstruct* OR impede* OR restrain* OR constrain* OR inhibit*)):ti,ab
#25	#5 AND #24
#26	[mh "Health Promotion"] OR ((vaccin* OR immuni*) NEAR/3 (equit* OR confidence OR complet* OR compliance OR comply OR complie* OR adher* OR access* OR facilitat* OR uptake OR "up take" OR "take up" OR improve* OR enhance* OR encourag* OR support* OR promot* OR optimiz* OR optimis* OR motivat* OR accept* OR incentive* OR attend* OR adopt* OR persuad* OR persuasion OR attitud* OR know* OR awareness* OR affordab*)):ti,ab
#27	#5 AND #26
#28	[mh "Physician Incentive Plans"] OR [mh "Reimbursement, Incentive"] OR [mh "Medical Audit"] OR [mh "Clinical Audit"] OR (incentive NEXT plan*):ti,ab OR reimbursement*:ti,ab OR (medical NEXT audit*):ti,ab OR (clinical NEXT audit*):ti,ab OR ((pediatric* OR parent* OR gp OR (general NEXT practic*) OR physician* OR doctor* OR clinician* OR (nurse NEXT practitioner*) OR midwife OR

Search No.	Search Terms
	midwives) NEAR/3 (attitud* OR know* OR awareness*)):ti,ab OR ((vaccin* OR immuni* OR parent* OR household* OR (house NEXT hold*) OR clinical) NEAR/3 "decision making"):ti,ab
#29	#5 AND #28
#30	[mh "Health Behavior"] OR [mh "Unsafe Sex"] OR ((unprotect* OR condomless OR risk OR "high risk" OR unsafe) NEAR/3 (sex* OR intercourse*)):ti,ab OR (sex* NEAR/2 (behavior* OR behaviour*)):ti,ab
#31	#5 AND #30
#32	[mh Alcoholism] OR alcoholism:ti,ab OR (alcohol* NEAR/2 (dependenc* OR addiction* OR abuse OR intoxication* OR use*)):ti,ab
#33	#5 AND #32
#34	[mh "Drug Misuse"] OR [mh "illicit drugs"] OR ((drug* OR substance* OR (non NEXT prescription*) OR nonprescription*) NEAR/2 (misuse OR abuse OR addiction* OR dependenc* OR use*)):ti,ab
#35	#5 AND #34
#36	[mh smoking] OR smoking:ti,ab OR smoker*:ti,ab
#37	#5 AND #36
#38	[mh Religion] OR religion*:ti,ab OR religious:ti,ab OR prayer*:ti,ab OR spirituality:ti,ab OR theology:ti,ab OR Mysticism:ti,ab OR buddhism:ti,ab OR christian*:ti,ab OR confucianism:ti,ab OR hinduism:ti,ab OR islam*:ti,ab OR judaism:ti,ab OR mormonism:ti,ab OR shamanism:ti,ab OR taoism:ti,ab
#39	#5 AND #38
#40	[mh "educational status"] OR (educational NEAR/3 status):ti,ab OR literacy:ti,ab OR illiteracy:ti,ab OR (academic NEAR/2 fail*):ti,ab
#41	#5 AND #40
#42	[mh "Social Justice"] OR [mh "Social Values"] OR [mh "Resource Allocation"] OR [mh "Health Care Rationing"] OR [mh "Health Priorities"/ES] OR equity:ti,ab
#43	#5 AND #42
#44	#7 OR #9 OR #11 OR #13 OR #15 OR #17 OR #19 OR #21 OR #23 OR #25 OR #27 OR #29 OR #31 OR #33 OR #35 OR #37 OR #39 OR #41 OR #43
#45	([mh animals] NOT [mh humans]) OR [mh "Animals, Laboratory"] OR [mh "Animal Experimentation"] OR [mh "Models, Animal"] OR [mh Rodentia] OR (rat OR rats OR mouse OR mice):ti
#46	(Comment OR letter OR editorial OR "Case Report" OR "conference avstract"):pt OR [mh news] OR [mh "historical article"] OR [mh "Anecdotes as Topic"] OR (letter OR comment*):ti
**Total**	
#47	#44 NOT (#45 OR #46)
#48	[mh "united states"] OR "united states" OR "usa" OR "u.s.a." OR ("u.s." NOT "Non-U.S.") OR alabama OR alaska OR arizona OR arkansas OR california OR colorado OR connecticut OR delaware OR florida OR georgia OR hawaii OR idaho OR illinois OR indiana OR iowa OR kansas OR kentucky OR louisiana OR maine OR maryland OR massachusetts OR michigan OR minnesota OR mississippi OR missouri OR montana OR nebraska OR nevada OR "new hampshire" OR "new jersey" OR "new mexico" OR "new york" OR "north carolina" OR "north dakota" OR ohio OR oklahoma OR oregon OR pennsylvania OR "rhode island" OR "south carolina" OR "south dakota" OR tennessee OR texas OR utah OR vermont OR virginia OR washington OR "west virginia" OR wisconsin OR wyoming OR "puerto rico"
#49	#47 AND #48<br>CDSR 4<br>CENTRAL 77

Table S-1d. PsychInfo Search Strategy (Conducted 10 June 2022)

Limits: Humans; English; No Comments, letters, editorials or dissertations. US only

Search No.	Search Terms
**Disease**	
S1	MA (Meningococcal Infections OR Neisseria meningitidis) OR TI (meningiti* OR meningococc* OR Micrococcus intracellularis OR Neisseria weichselbaumi OR IMD) OR AB (meningiti* OR meningococc* OR Micrococcus intracellularis OR Neisseria weichselbaumi OR IMD)
**Vaccination**	
S2	DE (Vaccination OR Immunization) OR MA (Vaccination OR Vaccines OR Immunization OR Immunization Programs) OR TI (vaccin* OR immuni*) OR AB (vaccin* OR immuni*)
S3	S1 AND S2
S4	MA Meningococcal Vaccines OR TI (acwy vax OR meningococcal vacci* OR antimeningococcal vacci* OR bexsero OR mc 4 vacci* OR mc4 vacci* OR MCP CRM197 conjugate vacci* OR men quad tt OR menactra OR menacwy OR menafrivac OR menbvac OR mencevax OR meningitec OR meningoccal vacci* OR meningococcal group B vacci* OR meningococcus vacci* OR meninvact OR menjugate OR menomune OR menpovax OR menquadfi OR menveo OR menzb OR neisvac c OR New Zealand candidate vacci* OR nimenrix OR nonamen OR tetramen-t OR trumenba OR va mengoc bc) OR AB (acwy vax OR meningococcal vacci* OR antimeningococcal vacci* OR bexsero OR mc 4 vacci* OR mc4 vacci* OR MCP CRM197 conjugate vacci* OR men quad tt OR menactra OR menacwy OR menafrivac OR menbvac OR mencevax OR meningitec OR meningoccal vacci* OR meningococcal group B vacci* OR meningococcus vacci* OR meninvact OR menjugate OR menomune OR menpovax OR menquadfi OR menveo OR menzb OR neisvac c OR New Zealand candidate vacci* OR nimenrix OR nonamen OR tetramen-t OR trumenba OR va mengoc bc)
S5	S3 OR S4
S6	DE Health Disparities OR MA (Health Equity OR Healthcare Disparities OR Health Status Disparities) OR TI ((health* N3 (disparit* or inequalit* or inequit* or equit* or equalit*))) OR AB ((health* N3 (disparit* or inequalit* or inequit* or equit* or equalit*)))
S7	S5 AND S6
S8	DE (Minority Groups OR Racial and Ethnic Groups OR Race and Ethnic Discrimination OR At Risk Populations OR Blacks OR African Cultural Groups OR American Indians OR Asians) OR MA (Minority Health OR "health disparity, minority and vulnerable populations" OR "ethnic and racial minorities" OR “hierarchy, social” OR social marginalization OR social vulnerability OR "Social Determinants of Health" OR African Americans OR African Continental Ancestry Group OR American Native Continental Ancestry Group OR Asian Continental Ancestry Group OR oceanic ancestry group) OR TI (Minority Health OR vulnerable* OR disadvantaged OR under served OR underserved OR neglect* OR marginal* OR socially excluded OR social deprivation OR social disparit* OR social determinant* OR social inequit* OR racial disparit* OR racial inequit* OR racial equalit* OR racial equity OR ethnic disparit* OR ((ethnic* OR minorit* OR race* OR racial) N3 (background OR communit* OR group* OR population*)) OR (high-risk N3 (communit* OR group* OR population* OR people OR person*)) OR african american* OR african ancestry OR aian OR asian* OR black* OR caucasian* OR ghetto* OR hispanic* OR indian* OR latino* OR latina* OR native american* OR pacific islander* OR people of color OR people of colour OR slum* OR white OR whites) OR AB (Minority Health OR vulnerable* OR disadvantaged OR under served OR underserved OR neglect* OR marginal* OR socially excluded OR social deprivation OR social disparit* OR social determinant* OR social inequit* OR racial

Search No.	Search Terms
	disparit* OR racial inequit* OR racial equalit* OR racial equity OR ethnic disparit* OR ((ethnic* OR minorit* OR race* OR racial) N3 (background OR communit* OR group* OR population*)) OR (high-risk N3 (communit* OR group* OR population* OR people OR person*)) OR african american* OR african ancestry OR aian OR asian* OR black* OR caucasian* OR ghetto* OR hispanic* OR indian* OR latino* OR latina* OR native american* OR pacific islander* OR people of color OR people of colour OR slum* OR white OR whites)
S9	S5 AND S8
S10	DE (Immigration OR Refugees) OR MA ("Emigrants and Immigrants" OR Refugees) OR TI (emigrant* OR Immigrant* OR migrant* OR refugee* OR Asylum Seeker* OR displaced person*) OR AB (emigrant* OR Immigrant* OR migrant* OR refugee* OR Asylum Seeker* OR displaced person*)
S11	S5 AND S10
S12	DE (Poverty OR poverty areas OR Socioeconomic Factors OR Homeless) OR MA (Poverty OR poverty area OR Medically Underserved Area OR socioeconomic factors OR Homeless Persons) OR TI (poverty OR low income* OR middle income* OR low resource* OR impoverish OR indigence OR indigency OR OR homeless OR jobless OR job seek* OR single parent* OR single mother* OR lone parent* OR health insurance status OR (socio economic* N3 (inequalit* OR unequal* OR inequit* OR disparit* OR imparit* OR discrepanc* OR imbalance* OR factor*))) OR AB (poverty OR low income* OR middle income* OR low resource* OR impoverish OR indigence OR indigency OR OR homeless OR jobless OR job seek* OR single parent* OR single mother* OR lone parent* OR health insurance status OR (socio economic* N3 (inequalit* OR unequal* OR inequit* OR disparit* OR imparit* OR discrepanc* OR imbalance* OR factor*)))
S13	S5 AND S12
S14	DE Gender Identity OR MA (“Sexual and Gender Minorities") OR TI (((Sexual OR Gender) N2 Minorit*) OR homosexual* OR homo sexual* OR bisexual* OR gay OR gays OR lesbian* OR pansexual* OR queer* OR asexual* OR transgender* OR transsexual* OR intersex* OR gender-expansive OR LGBT* OR GLBTI* OR 2 spirit* OR gender diverse OR gender fluid* OR non binary OR men who have sex with men) OR AB (((Sexual OR Gender) N2 Minorit*) OR homosexual* OR homo sexual* OR bisexual* OR gay OR gays OR lesbian* OR pansexual* OR queer* OR asexual* OR transgender* OR transsexual* OR intersex* OR gender-expansive OR LGBT* OR GLBTI* OR 2 spirit* OR gender diverse OR gender fluid* OR non binary OR men who have sex with men)
S15	S5 AND S14
S16	DE (Demographic Characteristics OR Community Resources OR Health Care Access OR Health Service Needs OR Rural Health OR Urban Health) OR MA (sociodemographic factors OR community resources OR Health Services Accessibility OR "Health Services Needs and Demand" OR health services, indigenous OR rural health OR rural health services OR urban health OR urban health services OR rural population OR urban population) OR TI (((geographic OR regional OR urban OR rural OR demographic OR cultural) N4 (inequalit* OR unequal* OR inequit* OR disparit* OR imparit* OR discrepanc* OR imbalance* OR barrier* OR facilitat* OR motivat*)) OR rural area* OR rural population* OR rural health* OR urban health* OR urban population*) OR AB (((geographic OR regional OR urban OR rural OR demographic OR cultural) N4 (inequalit* OR unequal* OR inequit* OR disparit* OR imparit* OR discrepanc* OR imbalance* OR barrier* OR facilitat* OR motivat*)) OR rural area* OR rural population* OR rural health* OR urban health* OR urban population*)
S17	S5 AND S16
S18	TI (((improv* OR enhanc* OR encourag* OR increas* OR support* OR assist* OR maximi* OR promot* OR facilitat*) N3 (uptake OR cover* OR acceptanc* OR complianc* OR complet* OR acceptab* OR adoption OR rate* OR access OR equit* OR equalit*))) OR AB (((improv* OR enhanc* OR encourag* OR increas* OR

Search No.	Search Terms
	support* OR assist* OR maximi* OR promot* OR facilitat*) N3 (uptake OR cover* OR acceptanc* OR complianc* OR complet* OR acceptab* OR adoption OR rate* OR access OR equit* OR equalit*)))
S19	S5 AND S18
S20	TI (((decreas* OR reduc* OR minimi* OR detect* OR chang*) N3 (inequalit* OR unequal* OR inequit* OR disparit* OR imparit* OR discrepanc* OR imbalance*))) OR AB (((decreas* OR reduc* OR minimi* OR detect* OR chang*) N3 (inequalit* OR unequal* OR inequit* OR disparit* OR imparit* OR discrepanc* OR imbalance*)))
S21	S5 AND S20
S22	DE Vaccination Attitudes OR MA Anti-Vaccination Movement OR TI (Anti-Vaccination OR anti-VAC OR anti-vaccine OR anti-vax* OR antivaccinal OR antivaccination OR antivaccine OR antivax*) OR AB (Anti-Vaccination OR anti-VAC OR anti-vaccine OR anti-vax* OR antivaccinal OR antivaccination OR antivaccine OR antivax*)
S23	S5 AND S22
S24	MA Vaccination Hesitancy OR TI (((vaccin* OR immuni*) N3 (hesitan* OR refus* OR delay* OR barrier* OR obstacle* OR restrict* OR obstruct* OR impede* OR restrain* OR constrain* OR inhibit*))) OR AB (((vaccin* OR immuni*) N3 (hesitan* OR refus* OR delay* OR barrier* OR obstacle* OR restrict* OR obstruct* OR impede* OR restrain* OR constrain* OR inhibit*)))
S25	S5 AND S24
S26	DE Health Promotion OR MA Health Promotion OR TI (((vaccin* OR immuni*) N3 (equit* OR confidence OR complet* OR compliance OR comply OR complie* OR adher* OR access* OR facilitat* OR uptake OR up take OR take up OR improve* OR enhance* OR encourag* OR support* OR promot* OR optimiz* OR optimis* OR motivat* OR accept* OR incentive* OR attend* OR adopt* OR persuad* OR persuasion OR attitud* OR know* OR awareness* OR affordab*))) OR AB (((vaccin* OR immuni*) N3 (equit* OR confidence OR complet* OR compliance OR comply OR complie* OR adher* OR access* OR facilitat* OR uptake OR up take OR take up OR improve* OR enhance* OR encourag* OR support* OR promot* OR optimiz* OR optimis* OR motivat* OR accept* OR incentive* OR attend* OR adopt* OR persuad* OR persuasion OR attitud* OR know* OR awareness* OR affordab*)))
S27	S5 AND S26
S28	DE Clinical Audits OR MA (Physician Incentive Plans OR "Reimbursement, Incentive" OR Medical Audit OR Clinical Audit) OR TI (incentive plan* OR reimbursement* OR medical audit* OR clinical audit* OR ((pediatric* OR parent* OR gp OR general practic* OR physician* OR doctor* OR clinician* OR nurse practitioner* OR midwife OR midwives) N3 (attitud* OR know* OR awareness*)) OR ((vaccin* OR immuni* OR parent* OR household* OR house-hold* OR clinical) N3 decision-making)) OR AB (incentive plan* OR reimbursement* OR medical audit* OR clinical audit* OR ((pediatric* OR parent* OR gp OR general practic* OR physician* OR doctor* OR clinician* OR nurse practitioner* OR midwife OR midwives) N3 (attitud* OR know* OR awareness*)) OR ((vaccin* OR immuni* OR parent* OR household* OR house-hold* OR clinical) N3 decision-making))
S29	S5 AND S28
S30	DE Health Behavior OR MA (Health Behavior OR Unsafe Sex) OR TI (((unprotect* OR condomless OR risk OR high-risk OR unsafe) N3 (sex* OR intercourse*)) OR (sex* N2 (behavior* OR behaviour*))) OR AB (((unprotect* OR condomless OR risk OR high-risk OR unsafe) N3 (sex* OR intercourse*)) OR (sex* N2 (behavior* OR behaviour*)))
S31	S5 AND S30
S32	DE Alcoholism OR MA Alcoholism OR TI (alcoholism OR (alcohol* N2 (dependenc* or addiction* or abuse or intoxication* or use*))) OR AB (alcoholism OR (alcohol* N2 (dependenc* or addiction* or abuse or intoxication* or use*)))
S33	S5 AND S32

Search No.	Search Terms
S34	DE Drug Abuse OR MA (Drug Misuse OR illicit drugs) OR TI (((drug* OR substance* OR non prescription* OR nonprescription*) N2 (misuse OR abuse OR addiction* OR dependenc* OR use*))) OR AB (((drug* OR substance* OR non prescription* OR nonprescription*) N2 (misuse OR abuse OR addiction* OR dependenc* OR use*)))
S35	S5 AND S34
S36	DE Tobacco Smoking OR MA smoking OR TI (smoking OR smoker*) OR AB (smoking OR smoker*)
S37	S5 AND S36
S38	DE Religion OR MA Religion OR TI (religion* OR religious OR prayer* OR spirituality OR theology OR Mysticism OR buddhism OR christian* OR confucianism OR hinduism OR islam* OR judaism OR mormonism OR shamanism OR taoism) OR AB (religion* OR religious OR prayer* OR spirituality OR theology OR Mysticism OR buddhism OR christian* OR confucianism OR hinduism OR islam* OR judaism OR mormonism OR shamanism OR taoism)
S39	S5 AND S38
S40	DE Academic Achievement OR MA educational status OR TI ((educational N3 status) OR literacy OR illiteracy OR (academic N2 fail*)) OR AB ((educational N3 status) OR literacy OR illiteracy OR (academic N2 fail*))
S41	S5 AND S40
S42	DE (Social Justice OR Social Values OR Resource Allocation) OR MA (Social Justice OR Social Values OR Resource Allocation OR Health Care Rationing OR Health Priorities) OR TI equity OR AB equity
S43	S5 AND S42
S44	S7 OR S9 OR S11 OR S13 OR S15 OR S17 OR S19 OR S21 OR S23 OR S25 OR S27 OR S29 OR S31 OR S33 OR S35 OR S37 OR S39 OR S41 OR S43
S45	((MA Animals NOT MA Humans)) OR MA (“Animals, Laboratory” OR Animal Experimentation OR “Models, Animal” OR Rodentia) OR TI (rat or rats or mouse or mice)
S46	PZ (letter OR editorial OR comment/reply OR Column/Opinion OR dissertation) OR TI (comment OR letter)
**Total**	
S47	S44 NOT (S45 OR S46)
S48	S44 NOT (S45 OR S46)<br>Limiters - English

Table S-1e. CINAHL Search Strategy (Conducted 31 May 2022)

Limits: Humans; English; No Comments, letters or editorials. US only

Search No.	Search Terms
**Disease**	
S1	MH "Meningococcal Infections+" OR SU (Meningococcal Infections OR Neisseria meningitidis) OR (TI (meningiti* OR meningococc* OR Micrococcus intracellularis OR Neisseria weichselbaumi OR IMD) OR AB (meningiti* OR meningococc* OR Micrococcus intracellularis OR Neisseria weichselbaumi OR IMD))
**Vaccination**	
S2	(MH "Immunization+") OR SU (Vaccination OR Vaccines OR Immunization OR Immunization Programs) OR (TI (vaccin* OR immuni*) OR AB (vaccin* OR immuni*))
S3	S1 AND S2
S4	(MH "Meningococcal Vaccines") OR SU Meningococcal Vaccines OR (TI (acwy vax OR meningococcal vacci* OR antimeningococcal vacci* OR bexsero OR mc 4 vacci* OR mc4 vacci* OR MCP CRM197 conjugate vacci* OR men quad tt OR menactra OR menacwy OR menafrivac OR menbvac OR mencevax OR meningitec OR meningoccal vacci* OR meningococcal group B vacci* OR meningococcus vacci* OR meninvact OR menjugate OR menomune OR menpovax OR menquadfi OR menveo OR menzb OR neisvac c OR New Zealand candidate vacci* OR nimenrix OR nonamen OR tetramen-t OR trumenba OR va mengoc bc) OR AB (acwy vax OR meningococcal vacci* OR antimeningococcal vacci* OR bexsero OR mc 4 vacci* OR mc4 vacci* OR MCP CRM197 conjugate vacci* OR men quad tt OR menactra OR menacwy OR menafrivac OR menbvac OR mencevax OR meningitec OR meningoccal vacci* OR meningococcal group B vacci* OR meningococcus vacci* OR meninvact OR menjugate OR menomune OR menpovax OR menquadfi OR menveo OR menzb OR neisvac c OR New Zealand candidate vacci* OR nimenrix OR nonamen OR tetramen-t OR trumenba OR va mengoc bc))
S5	S3 OR S4
S6	MH ("Healthcare Disparities" OR "Health Status Disparities") OR SU (Health Equity OR Healthcare Disparities OR Health Status Disparities) OR (TI ((health* N3 (disparit* or inequalit* or inequit* or equit* or equalit*))) OR AB ((health* N3 (disparit* or inequalit* or inequit* or equit* or equalit*))))
S7	S5 AND S6
S8	((MH "Minority Groups") OR (MH "Ethnic Groups+") OR (MH "Social Status") OR (MH "Social Determinants of Health") OR (MH "African Americans") OR (MH "Native Americans"+) OR (MH "Asians")) OR SU ((Minority Health OR "health disparity, minority and vulnerable populations" OR "ethnic and racial minorities" OR “hierarchy, social” OR social marginalization OR social vulnerability OR "Social Determinants of Health" OR African Americans OR African Continental Ancestry Group OR American Native Continental Ancestry Group OR Asian Continental Ancestry Group OR oceanic ancestry group)) OR (TI (Minority Health OR vulnerable* OR disadvantaged OR under served OR underserved OR neglect* OR marginal* OR socially excluded OR social deprivation OR social disparit* OR social determinant* OR social inequit* OR racial disparit* OR racial inequit* OR racial equalit* OR racial equity OR ethnic disparit* OR ((ethnic* OR minorit* OR race* OR racial) N3 (background OR communit* OR group* OR population*)) OR (high-risk N3 (communit* OR group* OR population* OR people OR person*)) OR african american* OR african ancestry OR aian OR asian* OR black* OR caucasian* OR ghetto* OR hispanic* OR indian* OR latino* OR latina* OR native american* OR pacific islander* OR people of color OR people of colour OR slum* OR white OR whites) OR AB (Minority Health OR vulnerable* OR disadvantaged OR under served OR underserved OR neglect* OR marginal* OR socially excluded OR social deprivation OR social disparit* OR social determinant* OR social inequit* OR racial

Search No.	Search Terms
	disparit* OR racial inequit* OR racial equalit* OR racial equity OR ethnic disparit* OR ((ethnic* OR minorit* OR race* OR racial) N3 (background OR communit* OR group* OR population*)) OR (high-risk N3 (communit* OR group* OR population* OR people OR person*)) OR african american* OR african ancestry OR aian OR asian* OR black* OR caucasian* OR ghetto* OR hispanic* OR indian* OR latino* OR latina* OR native american* OR pacific islander* OR people of color OR people of colour OR slum* OR white OR whites))
S9	S5 AND S8
S10	((MH "Emigration and Immigration") OR (MH "Refugees+")) OR SU ("Emigrants and Immigrants" OR Refugees) OR (TI (emigrant* OR Immigrant* OR migrant* OR refugee* OR Asylum Seeker* OR displaced person*) OR AB (emigrant* OR Immigrant* OR migrant* OR refugee* OR Asylum Seeker* OR displaced person*))
S11	S5 AND S10
S12	((MH "Poverty Areas") OR (MH "Poverty+") OR (MH "Medically Underserved Area") OR (MH "Socioeconomic Factors+") OR (MH "Homeless Persons")) OR SU (Poverty OR poverty area OR Medically Underserved Area OR socioeconomic factors OR Homeless Persons) OR (TI (poverty OR low income* OR middle income* OR low resource* OR impoverish OR indigence OR indigency OR OR homeless OR jobless OR job seek* OR single parent* OR single mother* OR lone parent* OR health insurance status OR (socio economic* N3 (inequalit* OR unequal* OR inequit* OR disparit* OR imparit* OR discrepanc* OR imbalance* OR factor*))) OR AB (poverty OR low income* OR middle income* OR low resource* OR impoverish OR indigence OR indigency OR OR homeless OR jobless OR job seek* OR single parent* OR single mother* OR lone parent* OR health insurance status OR (socio economic* N3 (inequalit* OR unequal* OR inequit* OR disparit* OR imparit* OR discrepanc* OR imbalance* OR factor*))))
S13	S5 AND S12
S14	((MH "Sexual and Gender Minorities+")) OR SU (“Sexual and Gender Minorities") OR (TI (((Sexual OR Gender) N2 Minorit*) OR homosexual* OR homo sexual* OR bisexual* OR gay OR gays OR lesbian* OR pansexual* OR queer* OR asexual* OR transgender* OR transsexual* OR intersex* OR gender-expansive OR LGBT* OR GLBTI* OR 2 spirit* OR gender diverse OR gender fluid* OR non binary OR men who have sex with men) OR AB (((Sexual OR Gender) N2 Minorit*) OR homosexual* OR homo sexual* OR bisexual* OR gay OR gays OR lesbian* OR pansexual* OR queer* OR asexual* OR transgender* OR transsexual* OR intersex* OR gender-expansive OR LGBT* OR GLBTI* OR 2 spirit* OR gender diverse OR gender fluid* OR non binary OR men who have sex with men))
S15	S5 AND S14
S16	((MH "Sociodemographic Factors") OR (MH "Community Resources") OR (MH "Health Services Accessibility+") OR (MH "Health Services Needs and Demand+") OR (MH "Health Services, Indigenous") OR (MH "Rural Health") OR (MH "Rural Health Services") OR (MH "Urban Health") OR (MH "Urban Health Services") OR (MH "Urban Population") OR (MH "Rural Population")) OR SU (sociodemographic factors OR community resources OR Health Services Accessibility OR "Health Services Needs and Demand" OR health services, indigenous OR rural health OR rural health services OR urban health OR urban health services OR rural population OR urban population) OR (TI (((geographic OR regional OR urban OR rural OR demographic OR cultural) N4 (inequalit* OR unequal* OR inequit* OR disparit* OR imparit* OR discrepanc* OR imbalance* OR barrier* OR facilitat* OR motivat*)) OR rural area* OR rural population* OR rural health* OR urban health* OR urban population*) OR AB (((geographic OR regional OR urban OR rural OR demographic OR cultural) N4 (inequalit* OR unequal* OR inequit* OR disparit* OR imparit* OR discrepanc* OR imbalance* OR barrier* OR facilitat* OR motivat*)) OR rural area* OR rural population* OR rural health* OR urban health* OR urban population*))
S17	S5 AND S16

Search No.	Search Terms
S18	TI (((improv* OR enhanc* OR encourag* OR increas* OR support* OR assist* OR maximi* OR promot* OR facilitat*) N3 (uptake OR cover* OR acceptanc* OR complianc* OR complet* OR acceptab* OR adoption OR rate* OR access OR equit* OR equalit*))) OR AB (((improv* OR enhanc* OR encourag* OR increas* OR support* OR assist* OR maximi* OR promot* OR facilitat*) N3 (uptake OR cover* OR acceptanc* OR complianc* OR complet* OR acceptab* OR adoption OR rate* OR access OR equit* OR equalit*)))
S19	S5 AND S18
S20	TI (((decreas* OR reduc* OR minimi* OR detect* OR chang*) N3 (inequalit* OR unequal* OR inequit* OR disparit* OR imparit* OR discrepanc* OR imbalance*))) OR AB (((decreas* OR reduc* OR minimi* OR detect* OR chang*) N3 (inequalit* OR unequal* OR inequit* OR disparit* OR imparit* OR discrepanc* OR imbalance*)))
S21	S5 AND S20
S22	(MH "Anti-Vaccination Movement") OR SU Anti-Vaccination Movement OR (TI (Anti-Vaccination OR anti-VAC OR anti-vaccine OR anti-vax* OR antivaccinal OR antivaccination OR antivaccine OR antivax*) OR AB (Anti-Vaccination OR anti-VAC OR anti-vaccine OR anti-vax* OR antivaccinal OR antivaccination OR antivaccine OR antivax*))
S23	S5 AND S22
S24	(MH "Vaccination Hesitancy") OR SU Vaccination Hesitancy OR (TI (((vaccin* OR immuni*) N3 (hesitan* OR refus* OR delay* OR barrier* OR obstacle* OR restrict* OR obstruct* OR impede* OR restrain* OR constrain* OR inhibit*))) OR AB (((vaccin* OR immuni*) N3 (hesitan* OR refus* OR delay* OR barrier* OR obstacle* OR restrict* OR obstruct* OR impede* OR restrain* OR constrain* OR inhibit*))))
S25	S5 AND S24
S26	(MH "Health Promotion+") OR SU Health Promotion OR (TI (((vaccin* OR immuni*) N3 (equit* OR confidence OR complet* OR compliance OR comply OR complie* OR adher* OR access* OR facilitat* OR uptake OR up take OR take up OR improve* OR enhance* OR encourag* OR support* OR promot* OR optimiz* OR optimis* OR motivat* OR accept* OR incentive* OR attend* OR adopt* OR persuad* OR persuasion OR attitud* OR know* OR awareness* OR affordab*))) OR AB (((vaccin* OR immuni*) N3 (equit* OR confidence OR complet* OR compliance OR comply OR complie* OR adher* OR access* OR facilitat* OR uptake OR up take OR take up OR improve* OR enhance* OR encourag* OR support* OR promot* OR optimiz* OR optimis* OR motivat* OR accept* OR incentive* OR attend* OR adopt* OR persuad* OR persuasion OR attitud* OR know* OR awareness* OR affordab*))))
S27	S5 AND S26
S28	((MH "Physician Incentive Plans") OR (MH "Reimbursement, Incentive") OR (MH "Audit")) OR SU (Physician Incentive Plans OR “Reimbursement, Incentive” OR Medical Audit OR Clinical Audit) OR (TI (incentive plan* OR reimbursement* OR medical audit* OR clinical audit* OR ((pediatric* OR parent* OR gp OR general practic* OR physician* OR doctor* OR clinician* OR nurse practitioner* OR midwife OR midwives) N3 (attitud* OR know* OR awareness*)) OR ((vaccin* OR immuni* OR parent* OR household* OR house-hold* OR clinical) N3 decision-making)) OR AB (incentive plan* OR reimbursement* OR medical audit* OR clinical audit* OR ((pediatric* OR parent* OR gp OR general practic* OR physician* OR doctor* OR clinician* OR nurse practitioner* OR midwife OR midwives) N3 (attitud* OR know* OR awareness*)) OR ((vaccin* OR immuni* OR parent* OR household* OR house-hold* OR clinical) N3 decision-making)))
S29	S5 AND S28
S30	((MH "Health Behavior+") OR (MH "Unsafe Sex")) OR SU (Health Behavior OR Unsafe Sex) OR (TI (((unprotect* OR condomless OR risk OR high-risk OR unsafe) N3 (sex* OR intercourse*)) OR (sex* N2 (behavior* OR behaviour*))) OR AB (

Search No.	Search Terms
	((unprotect* OR condomless OR risk OR high-risk OR unsafe) N3 (sex* OR intercourse*)) OR (sex* N2 (behavior* OR behaviour*))))
S31	S5 AND S30
S32	(MH "Alcoholism") OR SU Alcoholism OR (TI (alcoholism OR (alcohol* N2 (dependenc* or addiction* or abuse or intoxication* or use*))) OR AB (alcoholism OR (alcohol* N2 (dependenc* or addiction* or abuse or intoxication* or use*))))
S33	S5 AND S32
S34	SU (Drug Misuse OR illicit drugs) OR (TI (((drug* OR substance* OR non prescription* OR nonprescription*) N2 (misuse OR abuse OR addiction* OR dependenc* OR use*))) OR AB (((drug* OR substance* OR non prescription* OR nonprescription*) N2 (misuse OR abuse OR addiction* OR dependenc* OR use*))))
S35	S5 AND S34
S36	(MH "Smoking+") OR SU smoking OR (TI (smoking OR smoker*) OR AB (smoking OR smoker*))
S37	S5 AND S36
S38	((MH "Religion and Religions"+)) OR SU Religion OR (TI (religion* OR religious OR prayer* OR spirituality OR theology OR Mysticism OR buddhism OR christian* OR confucianism OR hinduism OR islam* OR judaism OR mormonism OR shamanism OR taoism) OR AB (religion* OR religious OR prayer* OR spirituality OR theology OR Mysticism OR buddhism OR christian* OR confucianism OR hinduism OR islam* OR judaism OR mormonism OR shamanism OR taoism))
S39	S5 AND S38
S40	(MH "Educational Status") OR SU educational status OR (TI ((educational N3 status) OR literacy OR illiteracy OR (academic N2 fail*)) OR AB ((educational N3 status) OR literacy OR illiteracy OR (academic N2 fail*)))
S41	S5 AND S40
S42	((MH "Social Justice+") OR (MH "Social Values+") OR (MH "Resource Allocation+") OR (MH "Health Priorities")) OR SU (Social Justice OR Social Values OR Resource Allocation OR Health Care Rationing OR Health Priorities) OR (TI equity OR AB equity)
S43	S5 AND S42
S44	S7 OR S9 OR S11 OR S13 OR S15 OR S17 OR S19 OR S21 OR S23 OR S25 OR S27 OR S29 OR S31 OR S33 OR S35 OR S37 OR S39 OR S41 OR S43
S45	((MH "Animals") NOT (MH "Human")) OR (MH "Animals, Laboratory") OR TI (rat or rats or mouse or mice)
S46	PT (letter OR editorial OR commentary) OR TI (comment OR letter)
**Total**	
S47	S44 NOT (S45 OR S46)
S48	(S44 NOT (S45 OR S46)) AND LA English
S49	TI ("united states" OR "usa" OR "u.s.a." OR "u.s." OR alabama OR alaska OR arizona OR arkansas OR california OR colorado OR connecticut OR delaware OR florida OR georgia OR hawaii OR idaho OR illinois OR indiana OR iowa OR kansas OR kentucky OR louisiana OR maine OR maryland OR massachusetts OR michigan OR minnesota OR mississippi OR missouri OR montana OR nebraska OR nevada OR "new hampshire" OR "new jersey" OR "new mexico" OR "new york" OR "north carolina" OR "north dakota" OR ohio OR oklahoma OR oregon OR pennsylvania OR "rhode island" OR "south carolina" OR "south dakota" OR tennessee OR texas OR utah OR vermont OR virginia OR washington OR "west virginia" OR wisconsin OR wyoming OR "puerto rico") OR AB ("united states" OR "usa" OR "u.s.a." OR "u.s." OR alabama OR alaska OR arizona OR arkansas OR california OR colorado OR connecticut OR delaware OR florida OR georgia OR hawaii OR idaho OR illinois OR indiana OR iowa OR kansas OR kentucky OR louisiana OR maine OR maryland OR massachusetts OR michigan OR minnesota OR mississippi OR missouri OR montana OR nebraska OR nevada OR "new hampshire" OR "new jersey" OR "new mexico" OR "new york" OR "north carolina" OR "north dakota" OR ohio OR oklahoma OR oregon

Search No.	Search Terms
	OR pennsylvania OR “rhode island” OR “south carolina” OR “south dakota” OR tennessee OR texas OR utah OR vermont OR virginia OR washington OR “west virginia” OR wisconsin OR wyoming OR “puerto rico”) OR MW (“united states” OR “usa” OR “u.s.a.” OR “u.s.” OR alabama OR alaska OR arizona OR arkansas OR california OR colorado OR connecticut OR delaware OR florida OR georgia OR hawaii OR idaho OR illinois OR indiana OR iowa OR kansas OR kentucky OR louisiana OR maine OR maryland OR massachusetts OR michigan OR minnesota OR mississippi OR missouri OR montana OR nebraska OR nevada OR “new hampshire” OR “new jersey” OR “new mexico” OR “new york” OR “north carolina” OR “north dakota” OR ohio OR oklahoma OR oregon OR pennsylvania OR “rhode island” OR “south carolina” OR “south dakota” OR tennessee OR texas OR utah OR vermont OR virginia OR washington OR “west virginia” OR wisconsin OR wyoming OR “puerto rico”) OR AF (“united states” OR “usa” OR “u.s.a.” OR “u.s.” OR alabama OR alaska OR arizona OR arkansas OR california OR colorado OR connecticut OR delaware OR florida OR georgia OR hawaii OR idaho OR illinois OR indiana OR iowa OR kansas OR kentucky OR louisiana OR maine OR maryland OR massachusetts OR michigan OR minnesota OR mississippi OR missouri OR montana OR nebraska OR nevada OR “new hampshire” OR “new jersey” OR “new mexico” OR “new york” OR “north carolina” OR “north dakota” OR ohio OR oklahoma OR oregon OR pennsylvania OR “rhode island” OR “south carolina” OR “south dakota” OR tennessee OR texas OR utah OR vermont OR virginia OR washington OR “west virginia” OR wisconsin OR wyoming OR “puerto rico”) Show Less
S50	S48 AND S49

Table S-2. PICOS Criteria

List of Criteria for the Inclusion and Exclusion of Studies During the Screening Process

Criteria	Included	Excluded
Population	▪ Studies that include individuals aged 10-25 years; individuals at increased risk of meningococcal infection	▪ Children aged < 10 years not at increased risk of meningococcal infection
Interventions and comparators	▪ MenACWY vaccines<br>– MenACWY-DT (Menactra)<br>– MenACWY-CRM (Menveo)<br>– MenACWY-TT (MenQuadfi)<br>▪ MenB vaccines<br>– MenB-FHbp (Trumenba)<br>– MenB-4C (Bexsero)	▪ Other meningococcal vaccines (e.g., Monovalent vaccines other than MenB: MenA, MenC, etc.)
Outcomes	▪ Health equity (e.g., access, receipt, and coverage of MenACWY or MenB vaccination)<br>▪ Vaccine awareness (ACWY or B)<br>▪ Vaccine knowledge (ACWY or B)<br>▪ Vaccine hesitancy (ACWY or B)<br>▪ Vaccine insurance coverage (ACWY or B)<br>▪ Physicians' awareness, knowledge, and attitude regarding meningococcal infection, availability of vaccines for serogroups ACWY and B, and category A or B recommendations for vaccines<br>▪ Patients' awareness of meningococcal disease and vaccines available for serogroups ACWY and B and their willingness to vaccinate their children against these infections	▪ Studies that do not report outcomes of interest in specified patient populations (i.e., individuals aged 10-25 years or individuals at increased risk of meningococcal infection)<br>▪ Outcomes for meningococcal vaccines other than MenACWY and MenB<br>▪ Outcomes not on the included list

Criteria	Included	Excluded
PROGRESS-Plus Factors[a]	▪ Place or state of residence (e.g., remote, overcrowding, homeless)<br>▪ Race (inclusive of all categories below)<br>– Ethnicity<br>– Culture<br>– Language<br>– Immigration<br>– Refugee status<br>▪ Occupation<br>▪ Gender identity or sex<br>▪ Religion or belief system<br>▪ Education or literacy<br>▪ Socioeconomic status<br>▪ Social capital (e.g., social support, networks, trust)<br>▪ Other<br>– Age<br>– Alcohol use or abuse<br>– Intravenous or illicit drug use<br>– Risk group (general)<br>– Sexual risk behaviors<br>– Smoking<br>– Preexisting conditions (e.g., disease, pregnancy)	▪ PROGRESS-Plus factors not on the included list
Study design	▪ Systematic reviews (including meta-analyses)<br>▪ Randomized controlled trials<br>▪ Observational studies, including phase 4 trials<br>▪ Cohort studies<br>▪ Cross-sectional studies<br>▪ Case-control studies<br>▪ Observational phase (e.g., long-term follow-up)	▪ Case reports<br>▪ Commentaries, letters, preprints, or editorials<br>▪ Preclinical studies
Limits	▪ English language<br>▪ No date limit	▪ Animal studies<br>▪ Non-US studies

MenA = serogroup A meningococcal; MenACWY = meningococcal conjugate; MenB = serogroup B meningococcal; MenC = serogroup C meningococcal; PICOS = Population and disease condition, interventions, comparators, outcomes, and study criteria; US = United States.

Note: If it is unclear whether a study meets any criterion during the level 1 screening process, the study will be progressed to full-text screening to confirm its inclusion in the review.

[a] PROGRESS is an acronym used by the Cochrane Equity Methods GroupNetwork to identify characteristics that stratify health opportunities and outcomes.

Table S-3. Summary of Study Design and Findings of Included Studies (n = 34)

No.	Reference/US geography (state)	Study design	Population/N	Objective	Quantitative measures reported (e.g., OR, RR)
1	Bart et al. [1]<br>Pennsylvania	A cross-sectional analysis of all 16- to 18-year-olds with a record in the Philadelphia immunization registry (KIDS Plus II) from 23 October 2015 through 31 July 2017, and the US Census American Community Survey (2016)	Parents of 16- to 18-year-olds<br>N = 85,489	To identify sociodemographic and provider factors associated with MenB vaccine receipt among 16- to 18-year-olds in Philadelphia county	Adjusted odds ratio
2	Basta et al. [2]<br>Minnesota	An in-person, cross-sectional survey at the University of Minnesota's Driven to Discover research facility during the 2017 Minnesota State Fair. Potential participants who approached the study booth were invited to take the survey.	Parents of children attending high school during the 2017-2018 academic year<br>N = 445	To determine how knowledgeable parents are about meningococcal vaccines and meningococcal disease and how willing they are to have their child vaccinated with MenB vaccines compared with MenACWY vaccine	Adjusted odds ratio
3	Bernstein et al. [3]<br>US, national	Retrospective analysis of state-level associations between voting patterns and adolescent coverage for at least 1 dose of HPV, Tdap, and MenACWY	Adolescents in the US who received HPV, MCV4, and/or tetanus-containing vaccination according to the 2012 NIS-Teen<br>N = Not provided	To examine state-level associations between voting patterns and adolescent coverage for at least 1 dose of HPV, tetanus-containing (Tdap), and MCV4	Kruskal-Wallis test
4	Cataldi et al. [4]<br>Colorado	Internet and mail survey of Colorado public health nurses, pediatric, and family medicine clinicians	Public health nurses, pediatric, and family medicine clinicians in Colorado<br>N = 163	To assess whether there were rural-urban differences in perceived logistic barriers to adolescent vaccination (Tdap, MenACWY, HPV, influenza),	Response rates

No.	Reference/US geography (state)	Study design	Population/N	Objective	Quantitative measures reported (e.g., OR, RR)
		from June to August 2019		perceived parental vaccine confidence and beliefs, and adolescent immunization delivery practices among Colorado HCPs	
5	Coyne-Beasley et al. [5]<br>North Carolina	A retrospective analysis of Carolina Behavioral Risk Factor Surveillance System (BRFSS) and Child Health Assessment and Monitoring Program (CHAMP) data to determine correlates of uptake of meningococcal Vaccine	2008 BRFSS caregivers (92% parents) of children aged 11-17 years in North Carolina who completed CHAMP survey<br>N = 1,281	To identify correlates of uptake of meningococcal vaccine as well as vaccine awareness among parents of adolescents in North Carolina. To describe reasons for nonvaccination among the parents who are aware of the vaccine.	Odds ratio
6	Darden et al. [6]<br>US, national	Computer-assisted telephone interview, survey analysis of teens examining reasons parents do not have their teens immunized	Adolescents 13-17 years old<br>Number of observations = 98,086	To examine reasons parents do not have their teens immunized and how these reasons have changed over time	Response rates
7	Dorell et al. [7]<br>US, national	Telephone survey of parents/guardians of adolescents 13-17 years of age	Parents/guardians of adolescents aged 13-17 years<br>N = 8,652	To describe factors that influence parental decisions to vaccinate their adolescent	Response rates
8	Gargano et al. [8]<br>Georgia	A telephone survey analysis designed to investigate demographic, behavioral, and psychosocial factors associated with parents' acceptance of vaccination for their adolescent children	Parents of middle and high school students<br>N = 114	To examine:<br>• Parent immunization attitudes<br>• Reasons for receipt or intention to have their adolescent receive recommended vaccines<br>• Importance of physician recommendations<br>• Psychosocial factors associated with receipt or intention to have	Response rates

No.	Reference/US geography (state)	Study design	Population/N	Objective	Quantitative measures reported (e.g., OR, RR)
				their adolescent receive a recommended vaccine	
9	Gowda and Dempsey [9] US, national	A cross-sectional study that used predictor variables including mandates, universal coverage policy, reimbursement levels, and other variables that were hypothesized to possibly confound the relationships between vaccine coverage levels and the state policies being studied	Adolescents aged 13-17 years enrolled in the 2009 NIS-Teen study<br>N = Not applicable	To examine associations between various state policies and coverage rates for 3 vaccines (Tdap, HPV, and MenACWY) recommended specifically for adolescents	Change in coverage rate
10	Gowda et al. [10] Michigan	A retrospective analysis of data from the Michigan Care Improvement Registry (MCIR; 2006-2010), a statewide immunization registry	Adolescents aged 11-18 years in Michigan<br>N = 1,252,655	To identify population-level clinical and demographic factors associated with adolescent vaccination	Adjusted odds ratio
11	Greenfield et al. [11] Washington	In-person surveys of Hispanic, Somali, and Ethiopian/Eritrean adolescents and parents of adolescents, and 3 focus groups with mothers of 11- to 18-year-olds were conducted to assess knowledge, attitudes, and barriers related to recommended adolescent vaccine	Hispanic (primarily Mexican), Somali, and Ethiopian/Eritrean parents and adolescents living in South King County (cities of Burien, SeaTac, and Tukwila), Washington<br>Adolescents: n = 45<br>Parents of adolescents: n = 157	To identify attitudes and knowledge of adolescent vaccination recommendations for Tdap, MCV4, and HPV vaccines among Hispanic, Somali, and Ethiopian/Eritrean communities in King County, Washington	Qualitative study; not applicable

No.	Reference/US geography (state)	Study design	Population/N	Objective	Quantitative measures reported (e.g., OR, RR)
12	Hansen and Niccolai [12]<br>US, national	Combined analysis of NIS-Teen data from 2017-2018 surveys (first 2 years for which NIS-Teen data on MenB were available)	Adolescents 17 years of age<br>N = 7,288	To determine patterns of MenB vaccine uptake following implementation of the 2015 ACIP recommendation for MenB vaccine receipt	Response rates
13	Healy et al. [13]<br>US, national	Analysis of NIS-Teen survey data (2012-2014) examining adolescent vaccination rates (≥ 1 dose Tdap vaccine, ≥ 1 dose MenACWY vaccine, ≥ 3 doses HPV vaccine) and for routine childhood vaccination catch-up doses (≥ 2 doses MMR vaccine, ≥ 2 doses varicella vaccine, and ≥ 3 doses HepB vaccine)	Adolescents aged 13-17 years<br>N = 58,090	To determine if disparities exist between foreign-born and US-born adolescents in vaccination coverage for Tdap, MenACWY, HPV, MMR, HepB, and varicella vaccines	Response rates
14	Huang et al. [14]<br>US, national	A web-based survey of US HCPs that explored characteristics associated with prescribing or receiving MenB and MenACWY vaccines, HCP knowledge of vaccine recommendations, and real-world practice pattern, in 2017	HCPs and patients aged 16-23 years of those providers who had received at least 1 childhood vaccination in the previous 6 months	(1) Examine HCP characteristics in relation to their prescribing patterns with meningococcal vaccines (MenACWY and MenB), (2) Understand real-world decision processes of HCPs and their interpretation of ACIP recommendations, and (3) assess patient characteristics associated with the receipt of MenB vaccines	Qualitative study; not applicable

No.	Reference/US geography (state)	Study design	Population/N	Objective	Quantitative measures reported (e.g., OR, RR)
15	Kempe et al. [15]<br>US, national	A multivariable analysis was conducted with the dependent variable of “always/almost always or often” initiating a discussion about MenB vaccine during routine well visits for adolescents aged 16-18 years	US pediatricians and family physicians	To assess among US pediatricians and family physicians:<br>▪ Practices regarding MenB vaccine delivery<br>▪ Factors influencing a decision to recommend vaccine<br>▪ Factors associated with discussing MenB vaccine at routine visits of 16- to 18-year-olds	Adjusted odds ratio
16	Kempe et al. [16]<br>US, national	A survey conducted via mail/e-mail between October and December 2016 among pediatricians and family physicians who were part of sentinel networks within each specialty	US Pediatricians and family practice doctors<br>N = 916	To assess among pediatricians and family physicians nationally (1) knowledge of the meaning of category A versus B recommendations and insurance coverage implications and (2) attitudes about category A and B recommendations	Adjusted odds ratio
17	Kurosky et al. [17]<br>US, national	Retrospective analysis of Commercial Claims and Encounters (CCAE) and Medicaid MarketScan Databases from 2011-2016 to determine receipt of 1 dose of MenACWY during early (10.5 through 13 years) and late (15.5 through 18 years) adolescence	Adolescents aged 10.5-18 years<br>CCAE: N = 796,639<br>Medicaid: N = 516,684	To identify factors associated with MenACWY uptake among adolescents. This study is complementary to the published NIS-Teen data.	Adjusted odds ratio

No.	Reference/US geography (state)	Study design	Population/N	Objective	Quantitative measures reported (e.g., OR, RR)
18	La et al. [18]<br>US, national	A cross-sectional, retrospective, exploratory analysis of preexisting anonymized 2017-2018 NIS-Teen data	Adolescents aged 17 years during 2017-2018<br>N = 7,288	To evaluate national- and regional-level ≥ 1 dose and ≥ 2 dose MenB vaccination coverage among US adolescents aged 17 years and to identify individual-level factors associated with MenB vaccination	Weighted odds ratio
19	Lu et al. [19]<br>US, national	Database analysis of 2015 NIS-Teen data	US NIS-Teen database, 2015<br>N = 21,875	To assess selected vaccination coverage among adolescents by health insurance status and other access-to-care characteristics	Adjusted prevalence ratios
20	Moss et al. [20]<br>US, national	Analysis of HCP-verified data from the 2010 NIS-Teen Survey for Tdap booster, meningococcal vaccine, and HPV vaccine (initiation among females)	Adolescents aged 13-17 years<br>N = 9,021	To examine the relationship of patient-driven versus provider-driven communication styles with vaccination	Response rates
21	Niccolai et al. [21]<br>US, national	Two-phase randomized phone survey followed by a medical record review in which HCPs complete an immunization grid	Adolescents 17 years of age<br>N = 3,807	To describe correlates of up-to-date MenACWY vaccination among adolescents aged 17 years in the US using data from NIS-Teen	Adjusted odds ratio
22	Packnett et al. [22]<br>US, national	Retrospective health insurance claims data analysis of individuals aged 16-23 years with a MenB vaccination during January 2017	Individuals aged 16-23 years<br>N = 213,162	To estimate and compare series completion rates for MenB-4C and MenB-FHbp during 2017-2020 among US individuals 16-23 years of age using the current dosing recommendations for MenB-FHbp.	Adjusted relative risk
23	Painter et al. [23]<br>Virginia	Semistructured interviews; purposive sample of 30 low-income, predominantly Latin-American	Low-income, uninsured, predominantly Latin-American immigrant mothers of adolescent	To assess the vaccine-related knowledge, attitudes, and decision-making for Tdap, MenACWY, and HPV among uninsured, low-	Qualitative study; not applicable

No.	Reference/US geography (state)	Study design	Population/N	Objective	Quantitative measures reported (e.g., OR, RR)
		immigrant mothers of adolescent daughters aged 13-17 years. Study participants were recruited from 2 academic-community managed health clinics in Virginia (March-September 2016).	daughters aged 13-17 years<br>N = 30	income, predominantly Latin-American immigrant mothers of adolescent daughters aged 13-17 years	
24	Phillips et al. [24]<br>Illinois	Longitudinal cohort study of young men who have sex with men using data collected from study U01DA036939 (RADAR)	Persons aged 16-29 years, assigned male at birth, English-speaking, and reported a sexual encounter with a male in the past year or identified as gay or bisexual<br>N = 486	To describe the knowledge and awareness of, and vaccination for, meningitis within a racially/ethnically diverse sample of young men who have sex with men (YMSM) in Chicago and to explore any racial differences that might explain disparities seen in the broader MSM community	Adjusted odds ratio
25	Pingali et al. [25]<br>Illinois	Retrospective analysis from the 2020 NIS-Teen database of adolescents aged 13-17 years	US NIS-Teen database, 2020	To estimate coverage with ACIP-recommended adolescent vaccines:<br>▪ Tdap, MenACWY, and HPV vaccines (11-12 years)<br>▪ Catch-up vaccination is recommended for HepB, HepA, MMR, and varicella vaccines for adolescents whose childhood vaccinations are not current<br>▪ Booster dose of MenACWY vaccine at age 16 years<br>▪ Shared clinical decision-making for	*t* tests using Taylor-series variance estimates

No.	Reference/US geography (state)	Study design	Population/N	Objective	Quantitative measures reported (e.g., OR, RR)
				the serogroup B meningococcal vaccine (MenB) for persons aged 16-23 years	
26	Pruitt et al. [26]<br>US, National	Retrospective analysis of adolescent vaccination coverage for 3 vaccines: Tdap, MenACWY, and HPV	Adolescents aged 13-17 years<br>N = 63,299	To describe the adolescent-, household-, and area-level characteristics associated with coverage patterns among all Tdap, MenACWY, and HPV in the US	Unadjusted and adjusted odds ratio
27	Rees-Clayton et al. [27]<br>Michigan	Retrospective analysis of 3 overlapping cohorts of 11-to 12-year-old children (n > 350,000 in each) to assess temporal trends in vaccination coverage, characteristics of those immunized, funding sources, and vaccination sites	Adolescents aged 11-12 years in Michigan<br>N = 1,081,542	To describe vaccine coverage of Michigan young adolescents immunized with tetanus, diphtheria, and pertussis; meningococcal conjugate; and human papillomavirus vaccines during 2006-2008	Odds ratio
28	Richardson et al. [28]<br>Florida	A cross-sectional analysis of survey data of MenB vaccine knowledge among parents and high-school students	Parents of 16- to 17-year-olds in Alachua County, Florida<br>N = 170	To identify rates and parent attitudes about MenB vaccination for 16- to 17-year-old adolescents	Qualitative study; not applicable
29	Seib et al. [29]<br>Georgia	A secondary cluster, randomized community trial among adolescent students enrolled in 11 schools in Georgia	Parents of middle- and high-school students attending 11 schools in Georgia<br>N = 686 parents	To examine the impact of a multifaceted intervention designed to promote vaccine acceptance among middle- and high-school adolescents in eastern Georgia	Odds ratio

No.	Reference/US geography (state)	Study design	Population/N	Objective	Quantitative measures reported (e.g., OR, RR)
30	Singer et al. [30]<br>US, national	A survey of insurance carriers in 47 states	Not applicable	To examine a recent national sample of private health insurance plans in the US for which benefits data were available and determine the frequency with which benefits for recommended childhood, adolescent, and adult vaccines are included	Qualitative study; not applicable
31	Srivastava et al. [31]<br>Pennsylvania	Online survey of US households	Adults with ≥ 1 dependent aged 16-19 years<br>N = 619	To evaluate the impact of the new school-entry vaccine requirements on clinical practice and vaccination rates among requirement-eligible adolescents within a large pediatric network in metropolitan Philadelphia	Odds ratio
32	Ghaswalla et al. [32]<br>US, national	A retrospective analysis of pooled 2016-2018 NIS-Teen data was conducted, including adolescents with adequate provider-reported vaccination data who were aged 17 years at the time of the survey	Adolescents aged 17 years in the US<br>N = 10,995	To identify factors associated with MenB vaccination, focusing on sociodemographic and healthcare resource use characteristics, as well as geographic region of residence	Adjusted odds ratio
33	Kricorian et al. [33]<br>Califorinia	A health literacy survey among adult female attendees of a health fair in an underserved area of Los Angeles	N = 43	To identify adult females familiarity with and recognition of specific words including: measles, shingles, pertussis, hepatitis, meningitis, stroke, diabetes, pneumonia, and HPV	Qualitative study; not applicable

No.	Reference/US geography (state)	Study design	Population/N	Objective	Quantitative measures reported (e.g., OR, RR)
34	Watkins and Feemster [34]<br>Not provided	A retrospective cohort study of 16- to 23-year-olds presenting to 31 primary care sites in a pediatric care network 23 October 2015 to 30 April 2017	16- to 23-year-olds<br>N = 45,428	To estimate the update of MenB after a category B recommendation	Response rates

ACIP = Advisory Committee on Immunization Practices; HCP = healthcare provider; HepA = hepatitis A; HepB = hepatitis B; HPV = human papillomavirus; MCV4 = meningococcal vaccine; MenACWY = meningococcal conjugate; MenB = serogroup B meningococcal; MMR = measles, mumps, and rubella; MSM = men who have sex with men; NIS-Teen = National Interview Survey-Teen; Tdap = tetanus, diphtheria, and pertussis; US = United States.

Table S-4. CASP Quality Assessments Results for Qualitative Studies (n = 6)

Source	CASP checklist questions									
	**1. Was there a clear statement of the aims of the research? (Yes, Can't tell, or No)**	**2. Is a qualitative methodology appropriate? (Yes, Can't tell, or No)**	**3. Was the research design appropriate to address the aims of the research?***	**4. Was the recruitment strategy appropriate to the aims of the research?***	**5. Was the data collected in a way that addressed the research issue?***	**6. Has the relationship between researcher and participants been adequately considered?***	**7. Have ethical issues been taken into consideration?***	**8. Was the data analysis sufficiently rigorous?***	**9. Is there a clear statement of findings?***	**10. How valuable is the research?***
Greenfield et al. [11]	Yes	Yes	Yes	Yes	Yes	Yes	Can't tell	Yes	Yes	Yes
Huang et al. [14]	Yes	Yes	Yes	Yes	Yes	Yes	Yes	Yes	Yes	Yes
Kricorian et al. [33]	Yes	Yes	Yes	Yes	Yes	Yes	Can't tell	Yes	Yes	Yes
Painter et al. [23]	Yes	Yes	Yes	Yes	Yes	Yes	Yes	Yes	Yes	Yes
Richardson et al. [28]	Yes	Yes	Yes	Yes	Yes	Yes	Yes	Yes	Yes	Yes
Singer et al. [30]	Yes	Yes	Yes	Yes	Yes	Yes	Yes	Yes	Yes	Yes

CASP = Critical Appraisal Skills Programme.
*Yes, No, Can’t tell.

Table S-5. CASP Quality Assessments Results for Cohort Studies (n = 28)

Source	Question													
	1. Did the study address a clearly focused issue?*	2. Was the cohort recruited in an acceptable way?*	3. Was the exposure accurately measured to minimize bias?*	4. Was the outcome accurately measured to minimize bias?*	5a. Have the authors identified all important confounding factors?*	5b. Have they taken account of the confounding factors in the design and/or analysis?*	6a. Was the follow-up of subjects complete enough?*	6b. Was the follow-up of subjects long enough?*	7. What are the results of this study? (e.g., ratio/rate difference, relative risk)	8. How precise are the results? (e.g., range of the confidence intervals, if given)	9. Do you believe the results?*	10. Can the results be applied to the local population?*	11. Do the results of this study fit with other available evidence?*	12. What are the implications of this study for practice?*
Bart et al. [1]	Yes	Yes	Can't tell	Can't tell	Can't tell	Yes	Can't tell	Can't tell	Adjusted odds ratio	Can't tell	Yes	No	Yes	Yes
Basta et al. [2]	Yes	Yes	Yes	Yes	Yes	Yes	NA	NA	Adjusted odds	95% CI reported, ranges of CIs vary	Yes	No	Yes	Yes
Bernstein et al. [3]	Yes	Yes	Yes	Yes	Yes	Yes	NA	NA	Wallis *t* test	95% CI reported, ranges of CIs vary	Yes	No	Yes	Yes
Cataldi et al. [4]	Yes	Yes	Yes	Yes	Can't tell	Can't tell	NA	NA	Response rates	Can't tell	Yes	No	Yes	Yes
Coyne-Beasley et al. [5]	Yes	Yes	Yes	Yes	Yes	Yes	NA	NA	Odds ratio	95% CI reported, ranges of CIs vary	Yes	No	No	Yes
Darden et al. [6]	Yes	Yes	No	Yes	No	Yes	NA	NA	Odds ratio	95% CI reported, ranges of CIs vary	Yes	No	Yes	Yes
Dorell et al. [7]	Yes	Yes	Yes	Yes	Yes	Yes	NA	NA	Odds ratio	95% CI reported, ranges of CIs vary	Yes	Yes	Yes	Yes

Source	Question													
	1. Did the study address a clearly focused issue?*	2. Was the cohort recruited in an acceptable way?*	3. Was the exposure accurately measured to minimize bias?*	4. Was the outcome accurately measured to minimize bias?*	5a. Have the authors identified all important confounding factors?*	5b. Have they taken account of the confounding factors in the design and/or analysis?*	6a. Was the follow-up of subjects complete enough?*	6b. Was the follow-up of subjects long enough?*	7. What are the results of this study? (e.g., ratio/rate difference, relative risk)	8. How precise are the results? (e.g., range of the confidence intervals, if given)	9. Do you believe the results?*	10. Can the results be applied to the local population?*	11. Do the results of this study fit with other available evidence?*	12. What are the implications of this study for practice?*
Gargano et al. [8]	Yes	Yes	Yes	Yes	Yes	No	Yes	Yes	"Fisher's exact test and chi-square tests"	Can't tell	Yes	No	Yes	Yes
Ghaswalla et al. [32]	Yes	Yes	Yes	Yes	Yes	Yes	Can't tell	Can't tell	Adjusted odds ratio	95% CI reported, ranges of CIs vary	Yes	No	Yes	Yes
Gowda and Dempsey [9]	Yes	Yes	Yes	Yes	Yes	Yes	NA	NA	Change in coverage rate	95% CI reported, ranges of CIs vary	Yes	No	Yes	Yes
Gowda et al. [10]	Yes	Yes	Yes	Yes	No	Yes	Yes	Yes	Adjusted odds ratio	95% CI reported, ranges of CIs vary	Yes	No	Yes	Yes
Hansen and Niccolai [12]	Yes	Yes	Yes	Yes	No	Yes	NA	NA	Unadjusted and adjusted odds ratio	% CI reported, ranges of CIs vary	Yes	No	Yes	Yes
Healy et al. [13]	Yes	Yes	Yes	Yes	No	Yes	NA	NA	Adjusted coverage prevalence ratio	% CI reported, ranges of CIs vary	Yes	No	Yes	Yes
Kempe et al. [15]	Yes	Yes	Yes	Yes	Yes	Yes	NA	NA	Risk ratio	95% CI reported, ranges of CIs vary	Yes	Yes	Yes	Yes
Kempe et al. [16]	Yes	Yes	Yes	Yes	Yes	Yes	NA	NA	Adjusted odds ratio	95% CI reported, ranges of CIs vary	Yes	No	Yes	Yes

Source	Question													
	1. Did the study address a clearly focused issue?*	2. Was the cohort recruited in an acceptable way?*	3. Was the exposure accurately measured to minimize bias?*	4. Was the outcome accurately measured to minimize bias?*	5a. Have the authors identified all important confounding factors?*	5b. Have they taken account of the confounding factors in the design and/or analysis?*	6a. Was the follow-up of subjects complete enough?*	6b. Was the follow-up of subjects long enough?*	7. What are the results of this study? (e.g., ratio/rate difference, relative risk)	8. How precise are the results? (e.g., range of the confidence intervals, if given)	9. Do you believe the results?*	10. Can the results be applied to the local population?*	11. Do the results of this study fit with other available evidence?*	12. What are the implications of this study for practice?*
Kurosky et al. [17]	Yes	Yes	Yes	Yes	Yes	Yes	NA	NA	Adjusted odds ratio	95% CI reported ranges of CIs vary	Yes	No	Yes	Yes
La et al. [18]	Yes	Yes	Yes	Yes	Yes	Yes	NA	NA	Odds ratio	95% CI reported, ranges of CIs vary	Yes	Yes	Yes	Yes
Lu et al. [19]	Yes	Yes	Yes	Yes	No	Yes	NA	NA	Adjusted prevalence ratios	95% CI reported, ranges of CIs vary	Yes	Yes	Yes	Yes
Moss et al. [20].	Yes	Yes	Yes	Yes	Yes	Yes	NA	NA	Odds ratio	95% CI reported, ranges of CIs vary	Yes	Yes	Yes	Yes
Niccolai et al. [21]	Yes	Yes	Yes	Yes	Yes	Yes	NA	NA	Adjusted odds ratio	95% CI reported, ranges of CIs vary	Yes	Yes	Yes	Yes
Packnett et al. [22]	Yes	Yes	Yes	Yes	Yes	Yes	Yes	Yes	Adjusted relative risk	95% CI reported, ranges of CIs vary	Yes	No	Yes	Yes
Phillips et al. [24]	Yes	Yes	Yes	Yes	Yes	Yes	NA	NA	Adjusted odds ratio	95% CI reported, ranges of CIs vary	Yes	No	Yes	Yes
Pingali et al. [25]	Yes	Yes	Yes	Yes	Yes	Yes	NA	NA	*t* tests using Taylor-series variance estimates were used to	95% CI reported, ranges of CIs vary	Yes	No	Yes	Yes

Source	Question													
	1. Did the study address a clearly focused issue?*	2. Was the cohort recruited in an acceptable way?*	3. Was the exposure accurately measured to minimize bias?*	4. Was the outcome accurately measured to minimize bias?*	5a. Have the authors identified all important confounding factors?*	5b. Have they taken account of the confounding factors in the design and/or analysis?*	6a. Was the follow-up of subjects complete enough?*	6b. Was the follow-up of subjects long enough?*	7. What are the results of this study? (e.g., ratio/rate difference, relative risk)	8. How precise are the results? (e.g., range of the confidence intervals, if given)	9. Do you believe the results?*	10. Can the results be applied to the local population?*	11. Do the results of this study fit with other available evidence?*	12. What are the implications of this study for practice?*
									assess vaccination coverage differences by survey year (2020 vs. 2019) and between socio-demographic groups					
Pruitt et al. [26]	Yes	Yes	Yes	Yes	Yes	Yes	NA	NA	Unadjusted and adjusted odds ratio	95% CI reported, ranges of CIs vary	Yes	No	Yes	Yes
Rees-Clayton et al. [27]	Yes	Yes	Yes	Yes	Yes	Yes	NA	NA	Odds ratio	95% CI reported, ranges of CIs vary	Yes	No	Yes	Yes
Seib et al. [29]	Yes	Yes	Yes	Yes	Yes	Yes	Yes	Yes	Odds ratio	95% CI reported, ranges of CIs vary	Yes	No	Yes	Yes
Srivastava et al. [31]	Yes	Yes	Yes	Yes	Yes	Yes	NA	NA	Odds ratio	95% CI reported, ranges of CIs vary	Yes	No	Yes	Yes

Source	Question														
	1. Did the study address a clearly focused issue?*	2. Was the cohort recruited in an acceptable way?*	3. Was the exposure accurately measured to minimize bias?*	4. Was the outcome accurately measured to minimize bias?*	5a. Have the authors identified all important confounding factors?*	5b. Have they taken account of the confounding factors in the design and/or analysis?*	6a. Was the follow-up of subjects complete enough?*	6b. Was the follow-up of subjects long enough?*	7. What are the results of this study? (e.g., ratio/rate difference, relative risk)	8. How precise are the results? (e.g., range of the confidence intervals, if given)	9. Do you believe the results?*	10. Can the results be applied to the local population?*	11. Do the results of this study fit with other available evidence?*	12. What are the implications of this study for practice?*
Watkins and Feemster [34]	Yes	Can't tell	Yes	Yes	Yes	Yes	Can't tell	Can't tell	Rates of MenACWY booster receipt	Can't tell	Yes	Can't tell	Yes	Yes

CASP = Critical Appraisal Skills Programme; CI = confidence interval; MenACWY = meningococcal conjugate; NA = not applicable.
*Yes, No, Can't tell.

Table S-6. Summary of Variation in Meningococcal Vaccine Coverage by Race/Ethnicity

Publication	Population, N	Vaccinated (MenACWY/MenB)	Results/Comparative Measure
Phillips et al. [24]	Young MSM<br>16-29 years old<br>N = 486	Meningococcal serogroup not reported: Self-reported: 49%	Vaccination coverage higher for White persons compared with Black persons<br>▪ Unadjusted OR: 2.02 (95% CI, 1.20-3.38; P < 0.05)<br>▪ Adjusted OR: 2.63 (95% CI, 1.49-4.64)
Pruitt et al. [26]	13- to 17-year-olds<br>N = 63,299<br>2015-2017	MenACWY (≥1 dose; parent/guardian reported):<br>Overall: 84.9%<br>▪ Black: 84.2%<br>▪ White: 81.7%	Vaccination coverage higher for non-Hispanic Black persons than non-Hispanic White persons
Niccolai et al. [21]	17-year-olds<br>N = 3,807	MenACWY (provider reported up to date[a]):<br>▪ White: 48.3%<br>▪ Black: 59.8%	Vaccination coverage higher for Black adolescents compared with White adolescents<br>▪ Unadjusted OR: 1.60 (95% CI, 1.15-2.22)<br>▪ Adjusted OR: 1.81 (95% CI, 1.26-2.60)<br>Vaccination coverage higher for Hispanic persons compared with White adolescents<br>▪ Unadjusted OR: 1.11 (95% CI, 0.78-1.60)<br>▪ Adjusted OR: 1.63 (95% CI, 1.11-2.39)
Coyne-Beasley et al. [5]	11-17 years<br>N =1,281	MenACWY (parent/guardian reported)<br>Of those who were aware of MenACWY (n=703): 44% (n = 280)	Vaccination coverage higher for non-Hispanic African Americans compared with non-Hispanic Whites<br>▪ Bivariate OR: 2.19 (95% CI, 1.32-3.63; P < 0.05)<br>▪ Multivariate OR: 2.17 (95% CI, 1.29-3.65; *P* < 0.05)
Kurosky et al. [17]	Younger: 10.5-13 years old<br>n=376,825<br>Older: 15.5-18 years old<br>n= 419,814	MenACWY (≥1 dose; claims data[b])<br>▪ Younger: 71.7%<br>▪ Older: 48.9%	Vaccination coverage higher for Black adolescents (N=189,879) versus White adolescents (N=227,156)<br>▪ Adjusted OR: 1.33 (95% CI, 1.31-1.35)
La et al. [18]	17-year-olds<br>N = 7,288	MenB vaccine ≥1 dose (provider reported), % (95% CI)<br>▪ Hispanic: 20.8 (16.4-26.1)<br>▪ Non-Hispanic White only: 13.5 (12.0-15.2)<br>▪ Non-Hispanic Black only: 16.1 (11.7-21.8)<br>▪ Non-Hispanic other/multiple races: 17.4 (11.8-24.8)	Vaccination coverage similar for non-Hispanic Black compared with non-Hispanic White (data controlled for demographic variables, insurance status, and previous vaccines)<br>▪ Adjusted OR: 0.74 (95% CI, 0.48-1.14)<br>No statistical difference between non-Hispanic Black and non-Hispanic White
Bart et al. [1]	16- to 18-year-olds<br>N = 85,489	MenB (≥1 dose)<br>▪ Black: 15.9%<br>Note: abstract data not provided for other races	Adolescents who reported unknown or other race were more likely than Black/African American to receive MenB<br>Unknown: Adjusted OR: 1.36<br>Other: Adjusted OR: 1.24<br>*P* < 0.0001 for both

Publication	Population, N	Vaccinated (MenACWY/MenB)	Results/Comparative Measure
Packnett et al. [22]	16-23 years old<br>N = 213,162<br>▪ Commercial: n = 156,080<br>▪ Medicaid: n = 57,082	MenB (series completion)<br>Black: (n = 21,748)<br>▪ MenB-4C: 42.9%<br>▪ MenB-FHbp: 30.8%<br>White: (n = 24,203)<br>▪ MenB-4C: 50.7%<br>▪ MenB-FHbp: 33.6%<br>Hispanic: (n = 4,909)<br>▪ MenB-4C: 50.0%<br>▪ MenB-FHbp: 41.5%<br>Other: (n = 6,165)<br>▪ MenB-4C: 52.6%<br>▪ MenB-FHbp: 37.2%	MenB series completion rates were lower among Black persons compared with White persons (Medicaid only) Adjusted RR: 0.86 (95% CI, 0.84-0.88)
Watkins and Feemster [34]	16-23 years old<br>N = 45,428	≥1 dose MenB, % (out of n)<br>▪ White: 27% (out of 26,280)<br>▪ Black: 18% (out of 13,186)<br>▪ Asian: 22% (out of 1,237)	Not applicable

CI = confidence interval; MSM = men who have sex with men; MenACWY = meningococcal conjugate; MenB = serogroup B meningococcal; MenB-FHbp = Trumenba®; MenB-4c = Bexsero®; OR = odds ratio; RR = rate ratio.
[a]Adolescents were up to date for the MenACWY vaccine if they received either 2 doses of MenACWY by age 17 or 1 dose at age 16 or 17 years.
[b] Commercial Claims and Encounters (CCAE) and MultiSite Medicaid Market Scan Research databases, maintained by Truven Health Analytics, LLC an IBM company (Truven Health).

Table S-7. Summary of differences in MenACWY and MenB coverage by Population Density

Publication	Population, N	Vaccinated (MenACWY/MenB)	Results/Qualitative Measure
Kurosky et al. [17]	Younger: 10.5-13 years<br>n=376,825<br>Older: 15.5-18 years<br>n= 419,814	MenACWY (≥1 dose; claims data[a])<br>Age: 10.5-13 years<br>▪ Rural: 58.21%<br>▪ Urban: 73.69%<br>▪ Unknown: 70.24%<br>MenACWY (≥ 1 dose; claims data[a])<br>Age: 15.5-18 years<br>▪ Rural: 34.24%<br>▪ Urban: 51.10%<br>▪ Unknown: 52.26%	Multivariate analysis showed lower coverage for rural residents compared with urban residents<br>Adjusted OR: 0.72 (95% CI, 0.71-0.73)
Pingali et al. [25]	13-17 years<br>N = 20,163	MenACWY (first dose; provider reported)<br>▪ Non-MSA: 85.7%<br>▪ MSA nonprincipal city: 89.4%<br>▪ MSA principal city: 90.2%<br>MenACWY (second dose; provider reported)<br>▪ Non-MSA: 50.1%<br>▪ MSA nonprincipal city: 58.5%<br>▪ MSA principal city: 50.6%	Only the coverages for first dose in non-MSAs and second dose in MSA nonprincipal cities were statistically significantly different from those in the other regions.
Pruitt et al. [26]	13- to 17-year-olds<br>N = 63,299	MenACWY (≥1 dose; parent/guardian reported):<br>▪ Overall: 82.8%<br>▪ Rural: 73.3%<br>▪ Urban: 83.9%	Coverage higher in urban areas than in rural
Bernstein et al. [3]	11-12 years old<br>N = not reported	MenACWY (median % coverage)<br>▪ Blue states (Democratic affiliation): 79.3%<br>▪ Red states (Republican affiliation): 72.8%	Coverage higher in blue states than in red states when the results were controlled for sociodemographics<br>Adjusted difference:14.1% (95% CI, 7.5%-21.0%; *P* <0.001)
Gowda et al. [10]	11-18 years<br>N = 1,252,655	MenACWY<br>▪ Urban: 48.9%<br>▪ Suburban: 47.9%<br>▪ Large/small town: 44.4%	Suburban and large/small town compared with urban residents<br>Adjusted OR: 0.95 (95% CI, 0.94-0.97)
Watkins and Feemster [34]	16-23 years old<br>N = 45,428	≥1 dose MenB, % (out of n)<br>▪ Urban: 21% (9,845)<br>▪ Suburban: 20% (35,583)	Not applicable

CI = confidence interval; MenACWY = meningococcal conjugate; MenB = serogroup B meningococcal; MSA = metropolitan statistical area; OR = odds ratio.
[a] Commercial Claims and Encounters (CCAE) and MultiState Medicaid Market Scan Research data, maintained by Truven Health Analytics, LLC and IBM company (Truven Health).

Table S-8. Estimated MenACWY vaccination coverage and doses among adolescents aged 13–17[a] years, by MSA[b] and by poverty level — National Immunization Survey-Teen, United States, 2020

	MSA, % (95% CI)[c]			Below poverty level, % (95% CI)[c]			At or above poverty level, % (95% CI)[c]		
Vaccine	Non-MSA (n = 3,678)	MSA nonprincipal city (n = 8,409)	MSA principal city (n = 8,076)	Non-MSA (n = 631)	MSA nonprincipal city (n = 865)	MSA principal city (n = 1,352)	Non-MSA (n = 2,938)	MSA nonprincipal city (n = 7,246)	MSA principal city (n = 6,420)
MenACWY[d]									
≥1 dose	85.7 (83.7–87.5)[e]	89.4 (87.9–90.7)	90.2 (88.7–91.5)	86.1 (81.8–89.5)[e]	87.2 (82.6–90.6)	91.6 (88.8–93.7)	85.6 (83.2–87.7)[e]	90.2 (88.6–91.5)	89.4 (87.5–91.0)
≥2 doses[f]	50.1 (43.4–56.9)	58.5 (54.0–62.8)[e]	50.6 (45.2–56.1)	47.4 (33.5–61.7)	47.6 (33.0–62.7)	48.6 (35.8–61.7)	50.2 (42.3–58.0)	61.2 (56.6–65.6)[e]	50.2 (44.1–56.4)

CI = confidence interval; MenACWY = meningococcal conjugate; MSA = metropolitan statistical area.
[a]Adolescents (n = 20,163) in the 2020 NIS-Teen were born during January 2002–January 2008.
[b]MSA status was determined based on household-reported county of residence and was grouped into 3 categories: MSA principal city, MSA nonprincipal city, and non-MSA. MSA and principal city were defined according to US Census Bureau (https://www.census.gov/programs-surveys/metro-micro.html). Non-MSA areas include urban populations not located within an MSA, as well as completely rural areas.
[c] Estimates with 95% CI widths >20 might not be reliable.
[d] Includes percentages receiving MenACWY and meningococcal-unknown type vaccine.
[e] Statistically significant difference ($P < 0.05$) in estimated vaccination coverage by metropolitan statistical area; referent group was adolescents living in MSA principal city areas.
[f] ≥2 doses of MenACWY or meningococcal-unknown type vaccine. Calculated only among adolescents who were aged 17 years at interview. Does not include adolescents who received 1 dose of MenACWY vaccine at age ≥16 years.
Source: Adapted from Pingali et al. [25].

Table S-9. Summary of Socioeconomic Status and MenACWY and MenB Vaccine Coverage

Publication	Population, N	Vaccinated (MenACWY/MenB)	Results/Comparative Measure, OR (95% CI)
Niccolai et al. [21]	17 years old<br>N = 3,807	MenACWY (provider reported up to date[a]):<br>Annual poverty level earnings<br>▪ Above poverty level and >$75,000: 50.7%<br>▪ Above poverty level but ≤$75,000: 47.3%<br>▪ Below poverty level: 54.6%	Annual poverty level earnings<br>▪ >$75,000 unadjusted OR: 0.75 (0.52-1.08)<br>▪ ≤$75,000 unadjusted OR: 0.86 (0.60-1.22)<br>Note: differences not statistically significant
Pingali et al. [25]	13-17 years old<br>N = 20,163	MenACWY (first dose; provider reported)<br>Below poverty level, at or above poverty level[b,c]<br>▪ Non-MSA: 86.1%, 85.6%<br>▪ MSA nonprincipal city: 87.2%, 90.2%<br>▪ MSA principal city: 91.6%, 89.4%<br>MenACWY (second dose; provider reported)<br>Below poverty level, at or above poverty level[b,c]<br>▪ Non-MSA: 47.4%, 50.2%<br>▪ MSA nonprincipal city: 47.6%, 61.2%<br>▪ MSA principal city: 48.6% 50.2%	Not estimated
Pruitt et al. [26]	13-17 years old<br>N = 63,299	MenACWY (≥1 dose; parent/guardian reported):<br>Compared with annual poverty level<br>▪ ≤$75,000: 80.4%<br>▪ >$75,000: 84.1%<br>▪ Below: 83.7%	Not estimated
La et al. [18]	17 years old<br>N = 7,288	MenB (≥1 dose; provider reported)<br>Compared with annual poverty level:<br>▪ >$75,000: 14.9%<br>▪ ≤$75,000: 14.1%<br>▪ Below: 19.6 (15.0-25.1)<br>No significant difference	Not estimated
Bart et al. [1]	16-18 years old<br>N = 85,489	MenB (≥1 dose)	Individuals in a neighborhood with median income >$100,000 were more likely to receive MenB than those in a neighborhood with median income < $20,000<br>Adjusted OR: 1.63; $P < 0.0001$

CI = confidence interval; MenACWY = meningococcal conjugate; MenB = serogroup B meningococcal; MSA = metropolitan statistical area; OR = odds ratio.
[a]Adolescent up to date for MenACWY if they either received 2 doses of MenACWY by age 17 years or 1 dose at age 16 or 17 years.
[b]Adolescents were classified as being below the federal poverty level if their total family income was less than the level specified for the applicable family size and number of children aged <18 years. All others were classified as at or above the poverty level (https://www.census.gov/data/tables/time-series/demo/income-poverty/historical-poverty-thresholds.html). Poverty status was unknown for 711 adolescents.
[c] MSA status was determined from household reported city and county of residence and was grouped into 3 categories: MSA principal city, MSA nonprincipal city, and non-MSA. MSA and MSA principal city were defined as by the US Census Bureau (https://www.census.gov/programs-surveys/metromicro. html). Non-MSAs include urban populations not located within an MSA and completely rural areas.

Table S-10. Summary of Studies Reporting Parent/Guardian Vaccine Awareness

Publication	Population, N	Aware of Meningitis/ Meningococcal disease, %	Aware of MenACWY and MenB, %	Aware of MenACWY, %	Aware of MenB, %	Vaccinated (MenACWY/MenB)
Basta et al. [2]	Parents of teens attending high school<br>N = 445	91.5%	75.5%	31.2%	20% Bexsero®<br>18% Trumenba®	Not applicable
Richardson et al. [28]	16- to 17-year-olds<br>N = 170	94%	Not reported	61%	40%	Of those who were aware of MenB<br>▪ 50% of parents reported their adolescent had been vaccinated<br>▪ 23% vaccination status was verified with state vaccination records
Coyne-Beasley et al. [5]	11- to 17-year-olds<br>N = 1,281	Not reported	Not applicable	65%<br>Note: Less likely to have heard of MenACWY if the children were Hispanic compared with non-Hispanic White children:<br>OR = 0.50, (95% CI, 0.28-0.89)	Not applicable	44% reported their children had been vaccinated with MenACWY
Srivastava et al. [31]	16- to 19-year-olds<br>N = 619	Not applicable	Not applicable	Not applicable	43%[a]<br>Race/ethnicity<br>▪ White, non-Hispanic: 60%<br>▪ Black, non-Hispanic: 12%<br>▪ Hispanic: 23%<br>▪ Other: 5%	Of those who were aware of MenB:<br>Parent reported vaccinated: 36%<br>Race/ethnicity (aware and vaccinated: n =161)<br>Race/ethnicity<br>▪ White, non-Hispanic: 56%<br>▪ Black, non-Hispanic: 15%<br>▪ Hispanic: 20%<br>▪ Other: 8%
Greenfield et al. [11]	11-18 years old<br>N = 157	Not applicable	Not applicable	33%<br>Bivariate analysis showed significant differences in MenACWY awareness among the 3 ethnic groups (*P* < 0.001)	Not applicable	Of those who were aware of MenACWY:<br>Parent reported vaccinated: 77%
Painter et al. [23]	13-17 years old<br>N = 30	50%	Not applicable	20%	Not applicable	Of the 30 total respondents: Parent-reported vaccinated: 16.7%

CI = confidence interval; MenACWY = meningococcal conjugate; MenB = serogroup B meningococcal; OR = odds ratio.
[a] Weighted % based on US population distribution.

Table S-11. MenB Vaccination Awareness by Race/Ethnicity and Vaccination Status (N = 619)

Race/ethnicity	Aware of MenB vaccines (n = 467)	Not aware of MenB vaccines (n = 155)	Aware of and vaccinated/intend to vaccinate (N = 312)	Aware of but do not intend to vaccinate (n = 155)	Aware of and vaccinated (n = 161)	Aware of and intend to vaccinate (n = 151)	Unaware of but interested (n = 52)	Unaware of but not interested (n = 103)
White, non-Hispanic	60%	42%	56%	65%	56%	55%	39%	56%
Black, non-Hispanic	12%	14%	14%	10%	15%	10%	12%	19%
Hispanic	23%	34%	22%	23%	20%	26%	39%	15%
Other	5%	10%	8%	2%	8%	8%	11%	11%

MenB = meningococcal B vaccine.
Note: All percentages in the table refer to weighted percentage.
Source: Adapted from Srivastava et al. [31].

Figure S-1. Meningococcal Disease on US College Campuses, 2013-2019

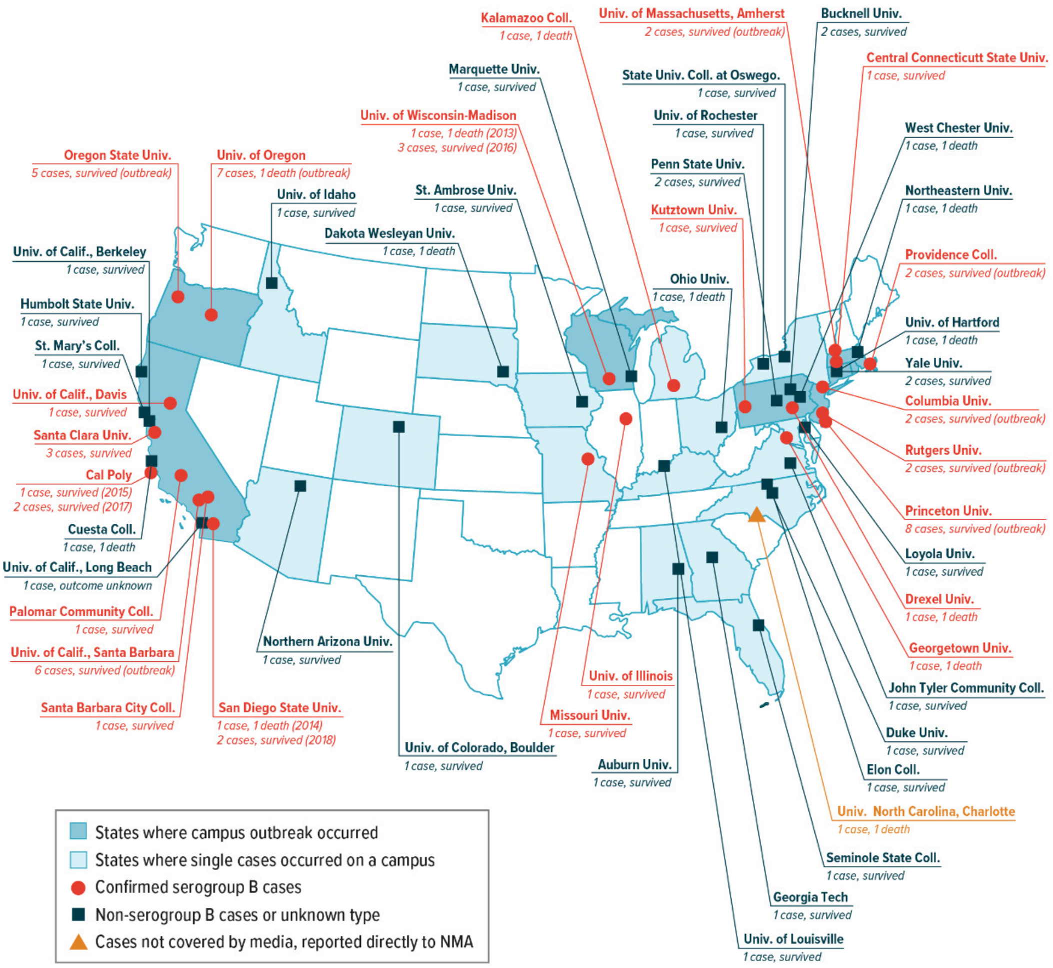

Source: National Meningitis Association [35].

REFERENCES

1. Bart S.M.; Eberhart M.; Feemster K. Impact of a Category B Recommendation: Meningococcal B (MenB) Vaccine Uptake Among Adolescents in Philadelphia County. 2018. Available online: https://plan.core-apps.com/pas2018/abstract/e5177900e60498db774eaebbee6695f0 (accessed on 19 July 2022).

2. Basta N.E.; Becker A.B.; Li Q.; Nederhoff D. Parental Awareness of Meningococcal B Vaccines and Willingness to Vaccinate Their Teens. Vaccine 2019, 37, 670-6. doi:http://dx.doi.org/https://dx.doi.org/10.1016/j.vaccine.2018.11.078.

3. Bernstein S.; North A.; Schwartz J.; Niccolai L.M. State-Level Voting Patterns and Adolescent Vaccination Coverage in the United States, 2014. Am J Pub Health 2016, 106, 1879-81. doi:http://dx.doi.org/https://dx.doi.org/10.2105/AJPH.2016.303381.

4. Cataldi J.R.; Brewer S.E.; Perreira C.; Furniss A.; Nederveld A.; Suresh K.; Williams C.; O'Leary S.T.; Dempsey A.F. Rural Adolescent Immunization: Delivery Practices and Barriers to Uptake. J Am Board Fam Med 2021, 34, 937-49. doi:http://dx.doi.org/https://dx.doi.org/10.3122/jabfm.2021.05.210107.

5. Coyne-Beasley T.; Reiter P.L.; Liberty A.C.; Ford C.A.; Miles D.R.; Brewer N.T. Awareness Is not Enough: The Need to Increase Meningococcal Vaccine Uptake. Clin Pediatr 2013, 52, 441-50. doi:http://dx.doi.org/https://dx.doi.org/10.1177/0009922813481847.

6. Darden P.M.; Thompson D.M.; Roberts J.R.; Hale J.J.; Pope C.; Naifeh M.; Jacobson R.M. Reasons for not Vaccinating Adolescents: National Immunization Survey of Teens, 2008-2010. Pediatrics 2013, 131, 645-51. doi:http://dx.doi.org/https://dx.doi.org/10.1542/peds.2012-2384.

7. Dorell C.; Yankey D.; Kennedy A.; Stokley S. Factors That Influence Parental Vaccination Decisions for Adolescents, 13 to 17 Years Old: National Immunization Survey-Teen, 2010. Clin Pediatr 2013, 52, 162-70. doi:http://dx.doi.org/https://dx.doi.org/10.1177/0009922812468208.

8. Gargano L.M.; Herbert N.L.; Painter J.E.; Sales J.M.; Morfaw C.; Rask K.; Murray D.; DiClemente R.J.; Hughes J.M. Impact of a Physician Recommendation and Parental Immunization Attitudes on Receipt or Intention to Receive Adolescent Vaccines. Human Vacc Immunother 2013, 9, 2627-33. doi:http://dx.doi.org/https://dx.doi.org/10.4161/hv.25823.

9. Gowda C.; Dempsey A.F. Medicaid Reimbursement and the Uptake of Adolescent Vaccines. Vaccine 2012, 30, 1682-9. doi:http://dx.doi.org/https://dx.doi.org/10.1016/j.vaccine.2011.12.097.

10. Gowda C.; Dong S.; Potter R.C.; Dombkowski K.J.; Dempsey A.F. A Population-Level Assessment of Factors Associated With Uptake of Adolescent-Targeted Vaccines in Michigan. J Adolesc Health 2013, 53, 498-505. doi:http://dx.doi.org/https://dx.doi.org/10.1016/j.jadohealth.2013.07.022.

11. Greenfield L.S.; Page L.C.; Kay M.; Li-Vollmer M.; Breuner C.C.; Duchin J.S. Strategies for Increasing Adolescent Immunizations in Diverse Ethnic Communities. J Adolesc Health 2015, 56, S47-53. doi:http://dx.doi.org/https://dx.doi.org/10.1016/j.jadohealth.2014.10.274.

12. Hansen C.E.; Niccolai L.M. Factors Associated With Receipt of Meningococcal B Vaccine Among United States Adolescents, National Immunization Survey-Teen, 2017-2018. J Adolesc Health 2021, 69, 769-73. doi:http://dx.doi.org/https://dx.doi.org/10.1016/j.jadohealth.2021.04.029.

13. Healy J.; Rodriguez-Lainz A.; Elam-Evans L.D.; Hill H.A.; Reagan-Steiner S.; Yankey D. Vaccination Coverage Among Foreign-Born and U.S.-Born Adolescents in the United States: Successes and Gaps – National Immunization Survey-Teen, 2012–2014. Vaccine 2018, 36, 1743-50. doi:http://dx.doi.org/10.1016/j.vaccine.2018.02.052.

14. Huang L.; Goren A.; Lee L.K.; Li V.W.; Dempsey A.; Srivastava A. Disparities in Healthcare Providers' Interpretations and Implementations of ACIP's Meningococcal Vaccine Recommendations. Hum Vaccin Immunother 2020, 16, 933-44. doi:http://dx.doi.org/https://dx.doi.org/10.1080/21645515.2019.1682845.

15. Kempe A.; Allison M.A.; MacNeil J.R.; O'Leary S.T.; Crane L.A.; Beaty B.L.; Hurley L.P.; Brtnikova M.; Lindley M.C.; Albert A.P. Adoption of Serogroup B Meningococcal Vaccine Recommendations. Pediatrics 2018, 142. doi:http://dx.doi.org/https://dx.doi.org/10.1542/peds.2018-0344.

16. Kempe A.; Allison M.A.; MacNeil J.R.; O'Leary S.T.; Crane L.A.; Beaty B.L.; Hurley L.P.; Brtnikova M.; Lindley M.C.; Liang J.L., et al. Knowledge and Attitudes Regarding Category B ACIP Recommendations Among Primary Care Providers for Children. Acad Pediatr 2018, 18, 763-8. doi:http://dx.doi.org/https://dx.doi.org/10.1016/j.acap.2018.04.005.

17. Kurosky S.K.; Esterberg E.; Irwin D.E.; Trantham L.; Packnett E.; Novy P.; Whelan J.; Hogea C. Meningococcal Vaccination Among Adolescents in the United States: A Tale of Two Age Platforms. The Journal of adolescent health : official publication of the Society for Adolescent Medicine 2019, 65, 107-15. doi:http://dx.doi.org/https://dx.doi.org/10.1016/j.jadohealth.2019.02.014.

18. La E.M.; Garbinsky D.; Hunter S.; Poston S.; Novy P.; Ghaswalla P. Meningococcal B Vaccination Coverage Among Older Adolescents in the United States. Vaccine 2021, 39, 2660-7. doi:http://dx.doi.org/https://dx.doi.org/10.1016/j.vaccine.2021.03.071.

19. Lu P.-J.; Yankey D.; Jeyarajah J.; O'Halloran A.; Fredua B.; Elam-Evans L.D.; Reagan-Steiner S. Association of Health Insurance Status and Vaccination Coverage Among Adolescents 13-17 Years of Age. J Pediatr 2018, 195, 256-62.e1. doi:http://dx.doi.org/https://dx.doi.org/10.1016/j.jpeds.2017.12.024.

20. Moss J.L.; Reiter P.L.; Rimer B.K.; Brewer N.T. Collaborative Patient-Provider Communication and Uptake of Adolescent Vaccines. Soc Sci Med 2016, 159, 100-7. doi:http://dx.doi.org/https://dx.doi.org/10.1016/j.socscimed.2016.04.030.

21. Niccolai L.M.; Yakely A.E.; Hansen C.E. Up-to-Date Coverage With Meningococcal Vaccine Among Adolescents Age 17 Years: Patterns and Correlates in the United States, 2017. Vaccine 2019, 37, 5934-8. doi:http://dx.doi.org/https://dx.doi.org/10.1016/j.vaccine.2019.08.015.

22. Packnett E.R.; Zimmerman N.M.; Kim G.; Novy P.; Morgan L.C.; Chime N.; Ghaswalla P. A Real-world Claims Data Analysis of Meningococcal Serogroup B Vaccine Series Completion and Potential Missed Opportunities in the United States. Pediatr Infect Dis J 2022, 41, e158-e65. doi:http://dx.doi.org/https://dx.doi.org/10.1097/INF.0000000000003455.

23. Painter J.E.; Viana De O Mesquita S.; Jimenez L.; Avila A.A.; Sutter C.J.; Sutter R. Vaccine-Related Attitudes and Decision-Making Among Uninsured, Latin American Immigrant Mothers of Adolescent Daughters: A Qualitative Study. Hum Vacc Immunother 2019, 15, 121-33. doi:http://dx.doi.org/https://dx.doi.org/10.1080/21645515.2018.1514353.

24. Phillips G., 2nd; Johnson A.K.; Adames C.N.; Mustanski B. Meningitis Vaccination, Knowledge, and Awareness Among YMSM in Chicago. Health Educ Behav 2018, 45, 607-15. doi:http://dx.doi.org/https://dx.doi.org/10.1177/1090198117752786.

25. Pingali C.; Yankey D.; Elam-Evans L.D.; Markowitz L.E.; Williams C.L.; Fredua B.; McNamara L.A.; Stokley S.; Singleton J.A. National, Regional, State, and Selected Local Area Vaccination Coverage Among Adolescents Aged 13-17 Years - United States, 2020. MMWR Morb Mortal Wkly Rep 2021, 70, 1183-90. doi:http://dx.doi.org/https://dx.doi.org/10.15585/mmwr.mm7035a1.

26. Pruitt S.L.; Tiro J.A.; Kepka D.; Henry K. Missed Vaccination Opportunities Among U.S. Adolescents by Area Characteristics. Am J Prevent Med 2022, 62, 538-47. doi:http://dx.doi.org/https://dx.doi.org/10.1016/j.amepre.2021.10.014.

27. Rees-Clayton E.; Montgomery J.P.; Enger K.S.; Boulton M.L. Trends in Michigan Early Adolescent Immunization: 2006-2008. Am J Pub Health 2012, 102, 1735-41. doi:http://dx.doi.org/https://dx.doi.org/10.2105/AJPH.2011.300577.

28. Richardson E.; Ryan K.A.; Lawrence R.M.; Harle C.A.; Young A.; Livingston M.D.; Rawal A.; Staras S.A.S. Perceptions and Knowledge About the MenB Vaccine Among Parents of High School Students. J Comm Health 2021, 46, 808-16. doi:http://dx.doi.org/https://dx.doi.org/10.1007/s10900-020-00954-1.

29. Seib K.; Underwood N.L.; Gargano L.M.; Sales J.M.; Morfaw C.; Weiss P.; Murray D.; Vogt T.M.; DiClemente R.J.; Hughes J.M. Preexisting Chronic Health Conditions and Health Insurance Status Associated With Vaccine Receipt Among Adolescents. J Adolesc Health 2016, 58, 148-53. doi:http://dx.doi.org/https://dx.doi.org/10.1016/j.jadohealth.2015.10.009.

30. Singer D.C.; Davis M.M.; Gebremariam A.; Clark S.J. Underinsurance for Recently Recommended Vaccines in Private Health Plans. J Community Health 2012, 37, 1164-7. doi:http://dx.doi.org/https://dx.doi.org/10.1007/s10900-012-9567-7.

31. Srivastava A.; Dempsey A.; Galitsky A.; Fahimi M.; Huang L. Parental Awareness and Utilization of Meningococcal Serogroup B Vaccines in the United States. BMC Public Health 2020, 20, 1109. doi:http://dx.doi.org/https://dx.doi.org/10.1186/s12889-020-09181-8.

32. Ghaswalla P.K.; Garbinsky D.; Poston S.; Hunter S.; Novy P.; La E.M. Correlates of and Disparities in Meningococcal B Vaccination Coverage Among 17-year-olds in the United States: A Pooled Analysis of 2016-2018 National Immunization Survey-Teen. Pediatrics 2021, 147, 296-8. doi:http://dx.doi.org/10.1542/peds.147.3-MeetingAbstract.296-a.

33. Kricorian K.; Lopez D.; Seu M.; Pham T.; Kigoonya R.; Equils O. The Role of Health Literacy in Vaccination Disparities: Do Patients Understand the Health Messages? Open Forum Infectious Diseases 2020, 7, S44. doi:http://dx.doi.org/10.1093/ofid/ofaa417.085.

34. Watkins E.; Feemster K. Factors Associated With Uptake of Meningococcus B Vaccination After an ACIP Category B Recommendation. Open Forum Infect Dis 2018, 5, S737.

35. National Meningitis Association. Meningococcal Disease on U.S. College Campuses, 2013-2019. 2019. Available online: https://nmaus.org/wp-content/uploads/2019/04/Alembic_NMA_Map_r28.pdf (accessed on 14 November 2022).
